# Evaluation of point-of-care multiplex polymerase chain reaction in guiding antibiotic treatment of patients acutely admitted with suspected community-acquired pneumonia in Denmark: A multicentre randomised controlled trial

**DOI:** 10.1371/journal.pmed.1004314

**Published:** 2023-11-28

**Authors:** Mariana Bichuette Cartuliares, Flemming Schønning Rosenvinge, Christian Backer Mogensen, Thor Aage Skovsted, Steen Lomborg Andersen, Claus Østergaard, Andreas Kristian Pedersen, Helene Skjøt-arkil

**Affiliations:** 1 Department of Emergency Medicine, University Hospital of Southern Denmark, Aabenraa, Denmark; 2 Department of Regional Health Research, University of Southern Denmark, Aabenraa, Denmark; 3 Department of Clinical Microbiology, Odense University Hospital, Odense, Denmark; 4 Research Unit of Clinical Microbiology, University of Southern Denmark, Odense, Denmark; 5 Department of Biochemistry and Immunology, University Hospital of Southern Denmark, Aabenraa, Denmark; 6 Department of Clinical Microbiology, University Hospital of Southern Denmark, Aabenraa, Denmark; 7 Department of Clinical Microbiology, Lillebaelt Hospital, Vejle, Denmark; 8 Department of Clinical Research, University Hospital of Southern Denmark, Aabenraa, Denmark; Erasme University Hospital, BELGIUM

## Abstract

**Background:**

Rapid and accurate detection of pathogens is needed in community-acquired pneumonia (CAP) to enable appropriate antibiotics and to slow the development of antibiotic resistance. We aimed to compare the effect of point-of-care (POC) polymerase chain reaction (PCR) detection of respiratory pathogens added to standard care with standard care only (SCO) on antibiotic prescriptions after acute hospital admission.

**Methods and findings:**

We performed a superiority, parallel-group, open-label, multicentre, randomised controlled trial (RCT) in 3 Danish medical emergency departments (EDs) from March 2021 to February 2022. Adults acutely admitted with suspected CAP during the daytime on weekdays were included and randomly assigned (1:1) to POC-PCR (The Biofire FilmArray Pneumonia Panel plus added to standard care) or SCO (routine culture and, if requested by the attending physician, target-specific PCR) analysis of respiratory samples. We randomly assigned 294 patients with successfully collected samples (tracheal secretion 78.4% or expectorated sputum 21.6%) to POC-PCR (*n* = 148, 50.4%) or SCO (146, 49.6%). Patients and investigators owning the data were blinded to the allocation and test results. Outcome adjudicators and clinical staff at the ED were not blinded to allocation and test results but were together with the statistician, blinded to data management and analysis. Laboratory staff performing standard care analyses was blinded to allocation. The study coordinator was not blinded. Intention-to-treat and per protocol analysis were performed using logistic regression with Huber–White clustered standard errors for the prescription of antibiotic treatment. Loss to follow-up comprises 3 patients in the POC-PCR (2%) and none in the SCO group. Intention-to-treat analysis showed no difference in the primary outcome of prescriptions of no or narrow-spectrum antibiotics at 4 h after admission for the POC-PCR (*n* = 91, 62.8%) odds ratio (OR) 1.13; (95% confidence interval (CI) [0.96, 1.34] *p* = 0.134) and SCO (*n* = 87, 59.6%). Secondary outcomes showed that prescriptions were significantly more targeted at 4-h OR 5.68; (95% CI [2.49, 12.94] *p* < 0.001) and 48-h OR 4.20; (95% CI [1.87, 9.40] *p* < 0.001) and more adequate at 48-h OR 2.11; (95% CI [1.23, 3.61] *p* = 0.006) and on day 5 in the POC-PCR group OR 1.40; (95% CI [1.18, 1.66] *p* < 0.001). There was no difference between the groups in relation to intensive care unit (ICU) admissions OR 0.54; (95% CI [0.10, 2.91] *p* = 0.475), readmission within 30 days OR 0.90; (95% CI [0.43, 1.86] *p* = 0.787), length of stay (LOS) IRR 0.82; (95% CI [0.63, 1.07] *p* = 0.164), 30 days mortality OR 1.24; (95% CI [0.32, 4.82] *p* = 0.749), and in-hospital mortality OR 0.98; (95% CI [0.19, 5.06] *p* = 0.986).

**Conclusions:**

In a setting with an already restrictive use of antibiotics, adding POC-PCR to the diagnostic setup did not increase the number of patients treated with narrow-spectrum or without antibiotics. POC-PCR may result in a more targeted and adequate use of antibiotics. A significant study limitation was the concurrent Coronavirus Disease 2019 (COVID-19) pandemic resulting in an unusually low transmission of respiratory virus.

**Trial registration:**

ClinicalTrials.gov (NCT04651712).

## Abstract 2

In this multicentre randomised controlled trial, Mariana Bichuette Cartuliares and colleagues evaluate point-of-care multiplex polymerase chain reaction in antibiotic treatment of patients acutely admitted with suspected community-acquired pneumonia in Denmark.

## Author summary

**Why was this study done?:**

- The global rise in antimicrobial resistance fueled by the excessive use and misuse of antibiotics is a major public health concern.
- Fast and accurate diagnostics is important to counteract this development as it can potentially reduce the use of antibiotics/broad-spectrum antibiotics without sacrificing patient safety.
- Pneumonia is a common, serious condition where available point-of-care (POC) technology (polymerase chain reaction) allows clinicians to detect possible airway pathogens before treatment decisions are made.

**What did the researchers do and find?:**

- In this randomised trial of 294 patients admitted with suspected pneumonia, POC did not result in the prescription of less antibiotics or less broad-spectrum antibiotics within 4 h after admission.
- Based on a subset of patients, the results indicated that more patients in the POC-group were treated with targeted or appropriate antibiotics 48 h and 5 days after admission.
- Patients in the POC-group had a non-statistically significant reduction in length of hospital stay of approximately 1 day.

**What do these findings mean?:**

- The use of respiratory POC does not seem to be an effective tool for reducing the use of antibiotics in a setting with a very low level of antimicrobial resistance and already prudent use of antibiotics.
- The use of respiratory POC may aid to ensure a targeted and/or appropriate treatment in a setting with a restrictive use of antibiotics—and thereby may aid to sustain a restrictive strategy.
- The concurrent Coronavirus Disease 2019 (COVID-19) pandemic and the unusually low transmission of common respiratory viruses in the period may have affected the results.

## Introduction

Community-acquired pneumonia (CAP) is a leading cause of hospitalisation and mortality [1,2]. Antibiotic treatment should be initiated timely [3] to avoid serious complications such as bacteremia, sepsis, organ failure, and death [4]. Initial antimicrobial treatment is often empiric, and an uncertain or delayed diagnosis often leads to use of broad-spectrum antibiotics [5]. This, in turn, contributes to adverse effects and complications, such as *Clostridioides difficile* infection, super-infections with resistant bacteria, poor patient outcomes, and general development of antibiotic resistance [6–9]. In Denmark antimicrobial resistance is low, and almost all *Streptococcus pneumoniae* are susceptible to benzylpenicillin and 93% to erythromycin, and 75% of *Haemophilus influenzae* are susceptible to benzylpenicillin [10]. Danish guidelines recommend narrow-spectrum penicillin for empirical treatment of CAP with CURB-65 <3 and broad-spectrum antibiotics for severe CAP with CURB-65 ≥3 [11,12]. The CAP diagnosis is based on clinical symptoms such as cough, dyspnea, fever, and sputum production, combined with unspecific diagnostic tools such as auscultation of the lungs, chest radiography, blood tests, and microbiological analysis of sputum samples [13–15].

Sputum samples can be cultivated to determine bacterial agents; however, samples are often of poor quality, many patients cannot deliver a sample and laboratory turnaround time is typically 2 days [16,17]. The lack of precise, timely microbiological results may delay or hinder targeted antimicrobial treatment.

In addition, CAP is often caused by viral infections that can be treated without antibiotics but usually are indistinguishable from bacterial infections without specific microbiological tests [18–20]. Consequently, molecular diagnostic methods, including rapid polymerase chain reaction (PCR) panels for viruses and bacteria, have been developed and tested in clinical settings [21–23]. These panels are simple to use, sensitive, generate rapid results, and significantly contribute to the management of CAP [21,23,24].

By identifying pathogenic organisms earlier, studies have reported faster de-escalation of antibiotic treatment, reduced duration of broad-spectrum empirical antibiotic therapy, reduced length of stay (LOS), and reduced hospital costs [25,26]. However, evidence of clinical impact of point-of-care (POC)-PCR testing of sputum samples in EDs is limited and a recent feasibility study advocates the need for randomised controlled trials (RCTs) to test POC-PCR panels in acute settings [27].

In this multicentre, randomised study, we aimed to investigate the effect of adding POC-PCR to standard care in an emergency department (ED) setting. Our hypothesis was that POC-PCR testing of sputum samples from suspected CAP patients would increase the proportion of patients treated with no or narrow-spectrum antibiotics. The objectives were (i) to investigate the effect of POC-PCR testing of sputum from suspected CAP patients on the prescriptions of antibiotic treatment compared to usual care; and (ii) to investigate if the addition of POC-PCR testing to the diagnostic setup affects LOS, intensive care unit (ICU) admission, 30-days mortality, in-hospital mortality, or readmissions within 30 days.

## Methods

### Trial design

This study was designed as a superiority, parallel-armed, multicentre randomised controlled clinical trial, and was part of a large multifaceted clinical study “**IN**fectious **D**iseases in **E**m**E**rgency **D**epartment” (INDEED) [28].

The study was reported in accordance with the Consolidation Standard of Reporting Trials (CONSORT) guidelines (see S1 Text) [29]. The processing of personal data is notified to and approved by the Region of Southern Denmark and listed in the internal record (no. 20/60508) cf. Art 30 of The EU General Data Protection Regulation and approved by the Regional Committee on Health Research Ethics for Southern Denmark (S-20200188), registered by ClinicalTrials.gov (NCT04651712), and conducted according to the Declaration of Helsinki-Ethical principle for medical research involving human subjects. The study protocol (see S2 Text) has been published and includes further information about the methods [28].

### Setting

The trial was conducted in 3 Danish medical EDs with a coverage of approximately 750.000 inhabitants: 2 regional hospitals, Lillebælt Hospital in Kolding and Hospital Sønderjylland in Aabenraa, and 1 university hospital, Odense University Hospital in Odense. Based on data from the National Health Data Agency and Statistics Denmark, the mean hospital LOS for patients >65 years old hospitalised in departments with medical specialties (including pneumonia) was of 5.9 days in 2018 [30], and local data from the 3 hospitals included in this study, reported a mean LOS of 3.8 days in hospital for adult patients (>18 years) discharged with pneumonia diagnose during the study period. According to clinical guidelines, patients admitted to the ED in our institutions must have a clinical assessment within half an hour to clarify suspicion of infection and disease severity. If the ED physician suspects CAP, diagnostic biomarkers, chest X-ray, and tracheal suctioning/aspirates, or expectorated sputum are performed without delay [11,12]. If indicated, empirical treatment must be initiated within 4 h, and the treatment must be documented in the patient medical chart. The empirical treatment guidelines for CAP are presented in S1 Table, and the timeline for the standard procedures in the EDs is presented in S2 Table.

### Participants

Adults aged 18 years or older admitted to the ED were invited to participate in the study if the attending physician suspected CAP and the patient had at least one of the following pulmonary symptoms: dyspnea, cough, expectoration, chest pain, or fever. Patients were excluded if: they could not deliver a sputum sample, participation delayed urgent treatment, the patient was transferred to an ICU, the patient had been admitted within the last 14 days, had Coronavirus Disease 2019 (COVID-19) infection at admission, was pregnant, or had severe immunodeficiencies (HIV–positive, with a cluster of differentiation 4 cell count <200), treatment with immunosuppressive medicine (Anatomical Therapeutic Chemical classification L04A), corticosteroids (>20 mg/day prednisone or equivalent for >14 days within the last 30 days), or chemotherapy within 30 days [28]. If patients fulfilled the eligibility criteria, the study assistant obtained verbal and written consent (see S3 Text) which was documented and witnessed at the bedside immediately after clinical assessment and before inclusion in the study. Patients were recruited consecutively Monday through Friday from 10 AM to 8 PM.

### Randomisation and masking

The patient was randomly assigned to one of 2 groups with 1:1 allocation: (i) POC-PCR analysis (Biofire FilmArray Pneumonia Panel plus, Biomérieux, Marcy l’Etoile, France) [31] in addition to standard care; or (ii) standard care only (SCO) as control. The randomisation was generated electronically using Research Electronic Data Capture Randomisation Module [32]. Computer-generated random lists were prepared by an independent data manager with permuting blocks of varying size and stratified according to sites. Allocation concealment was ensured, as randomisation was performed electronically, and the study assistants administering the randomisation did not have access to the randomisation code. The allocation was not revealed to the project assistant before consent was obtained and specimen collected. Patients and investigators owning the data were blinded to the allocation and test results. Outcome adjudicators and clinical staff at the ED were not blinded to allocation and test results but were together with the statistician, blinded to data management and analysis. Laboratory staff performing standard care analyses was blinded to allocation. The study coordinator was not blinded.

### Procedure

Tracheal secretion is the recommended sampling method by Danish national and regional guidelines [11,12], but expectorated sputum is accepted if the patient can not cooperate during the procedure. Lower respiratory tract (LRT) specimens were collected right after enrolment by a project assistant. Tracheal suction/aspiration was performed with a catheter (EXTRUDAN Surgery Aps, Denmark, CH12, 530 mm) insertion into the nares during inhalation. The catheter was gently advanced about 40 cm into the trachea, where suctioning at 200 to 400 mmHg was performed before withdrawing the catheter. POC-PCR analysis was done without delay in a POC laboratory. The POC laboratory had 24-h coverage and was situated in the ED (2 sites) or close to the department (transport time less than 10 min, 1 site). Project assistants and laboratory staff were trained in the use of the POC-PCR system, and each site had a pocket laboratory protocol to ensure sample quality and safe handling of specimens. Within 4 h after the patient was admitted, the result of the POC-PCR was handed to the treating physician along with a guideline-based action card (see S4 Text) recommending specific treatments matching different POC-PCR results. In case of any additional questions, the physician was encouraged to contact the local clinical microbiologist for further advice. All 6 project assistants received bedside training in tracheal suction to ensure consistent data collection. Clinical and patient data were retrieved by chart review and patient interview as described in the protocol [28].

### Intervention

#### Point-of-care polymerase chain reaction (POC-PCR)

The Biofire FilmArray Pneumonia Panel plus (Biomérieux, Marcy l’Etoile, France) is an automatic, closed, multiplex PCR, that includes all steps of molecular diagnostics in about 75 min, including sample preparation. The panel detects 18 bacterial pathogens, 9 viruses, and 7 antimicrobial resistance genes (see S3 Table).

Results for typical colonising bacteria were reported semiquantitatively providing estimates to the nearest whole log as gene copies/ml ranging from 10^4^ to 10^7^ copies/ml. Biofire FilmArray Pneumonia Panel was used in accordance with the manufacturer’s instructions at all 3 sites [31]. All POC-PCR results were registered directly in a study database and in the patient’s medical chart.

#### Standard care (routine culture and PCR)

All samples were submitted to standard-of-care procedures of microbiological testing. Part of the sputum sample was transferred to a 5% blood agar plate and to a chromogenic and/or selective agar. The inoculum was streaked over the agar surface and blood agar plates were inoculated with a *Staphylococcus* streak to allow growth of *H*. *influenzae*. Blood agar plates were incubated in a 5% CO2 atmosphere, other plates at 35°C in normal atmospheric conditions. After 1 to 2 days of incubation, pathogens were identified by Matrix-Assisted Laser Desorption/Ionisation-time of flight and reported semiquantitatively as few, some, or numerous. In addition, “no growth of pathogens” and “upper airway microbiota” were reported. Routine PCR was performed if requested by the referring physician (e.g., for *Legionella pneumophila* or influenza virus). The results were registered in the microbiological laboratory information system (MADS, Aarhus University Hospital, Aarhus, Denmark) and were accessible from the patient’s medical chart.

### Outcomes

The primary outcome was the prescription of “no or narrow-spectrum” antibiotics within 4 h after admission. Narrow-spectrum antibiotics were defined as antibiotics active against CAP pathogens: Beta-lactamase sensitive penicillins (phenoxymethylpenicillin or benzylpenicillin), extended spectrum beta-lactamase sensitive penicillins (ampicillin/amoxicillin/pivampicillin). In case of penicillin allergy: macrolides and cefuroxime were also defined as narrow-spectrum antibiotics (see S4 Table). We pooled narrow-spectrum and no antibiotics, as our focus was rational and restrictive use of antibiotics [11,12]. As our main focus was to study POC-PCR from an antibiotic stewardship perspective, we decided to handle no and narrow-spectrum antibiotics as our primary outcome and targeted antibiotics as a secondary outcome. In the initial protocol, no, narrow-spectrum, and targeted antibiotics were treated as a composite primary outcome [28].

### Secondary outcomes

- Prescription of no or narrow-spectrum antibiotics at 48 h and 5 days after admission.
- Prescription of targeted antibiotics within 4 h, 48 h, and 5 days. Targeted antibiotics were defined as either narrow-spectrum antibiotics targeting CAP or antibiotics directed against a detected bacterial pathogen identified by culture.
- Prescription of adequate antibiotics within 4 h, 48 h, and 5 days. Adequate antibiotics were defined as all antibiotics covering the detected bacterial pathogen.

We categorised antibiotic treatment as targeted and/or adequate in relation to the following pathogens identified by culture: *S*. *pneumoniae*, *H*. *influenzae*, *Moraxella catarrhalis*, *Pseudomonas aeruginosa*, *Staphylococcus aureus*, hemolytic streptococci, and *L*. *pneumophila* (see S5 Table). We excluded *Enterobacterales*, *Acinetobacter*, and yeast as they usually represent colonisation and are less likely to cause CAP.

Data on other secondary outcomes were extracted from the patients’ medical chart: 30 days mortality (death within 30 days from admission to the ED), in-hospital mortality (death during the current hospitalisation, ICU admission during the current hospitalisation, readmission within 30 days after discharge and LOS (days from admission to discharge)).

### Statistical methods

Based on literature and local data, we assumed that adherence to antimicrobial guidelines was 50% for the management of CAP patients [33], and we required at least 200 patients with suspected CAP with two-sided 5% significance to achieve a power of 82% to detect a minimal difference of 20% prescription of no or narrow-spectrum treatment in the POC-PCR group compared to the control group. However, more patients were included, so the power calculation was repeated without changing earlier assumptions before the commencement of statistical analysis and with the statistician blinded to the allocation groups and the general distribution of the data. The new calculation yielded a power of 94% with 290 patients with two-sided 5% significance.

Descriptive statistics were conducted to assess whether the exchangeability assumption was met for the baseline variables. To assess whether there was a difference between the 2 groups Fisher’s exact test or chi-square test were performed for categorical variables, and *t* test or Wilcoxon rank sum test for non-categorical variables.

To accommodate the variation between study sites, we used logistic regression with Huber–White clustered standard errors to investigate the effect of POC-PCR on antibiotic prescription at 4 h, for the secondary outcomes at 48 h, and 5 days. Results were reported with odds ratio (OR) and 95% confidence intervals (CIs). To compare the 2 groups, we used negative binomial regression for LOS. Logistic regression analyses were performed for 30 days mortality, in-hospital mortality, ICU admission, and readmission within 30 days and unadjusted and adjusted for triage. Multiple imputation using a logistic regression was performed to handle missing outcome data. We realised 50 imputations of target treatment at 4 h based on CURB-65 and age as they may be good predictors for antibiotic treatment prescriptions. We considered a two-sided *p*-value less than 0.05 statistically significant, and no adjustments for multiple testing were utilised. Statistical analyses were performed using STATA 17.0 (Texas, United States of America).

## Results

**Fig 1:**
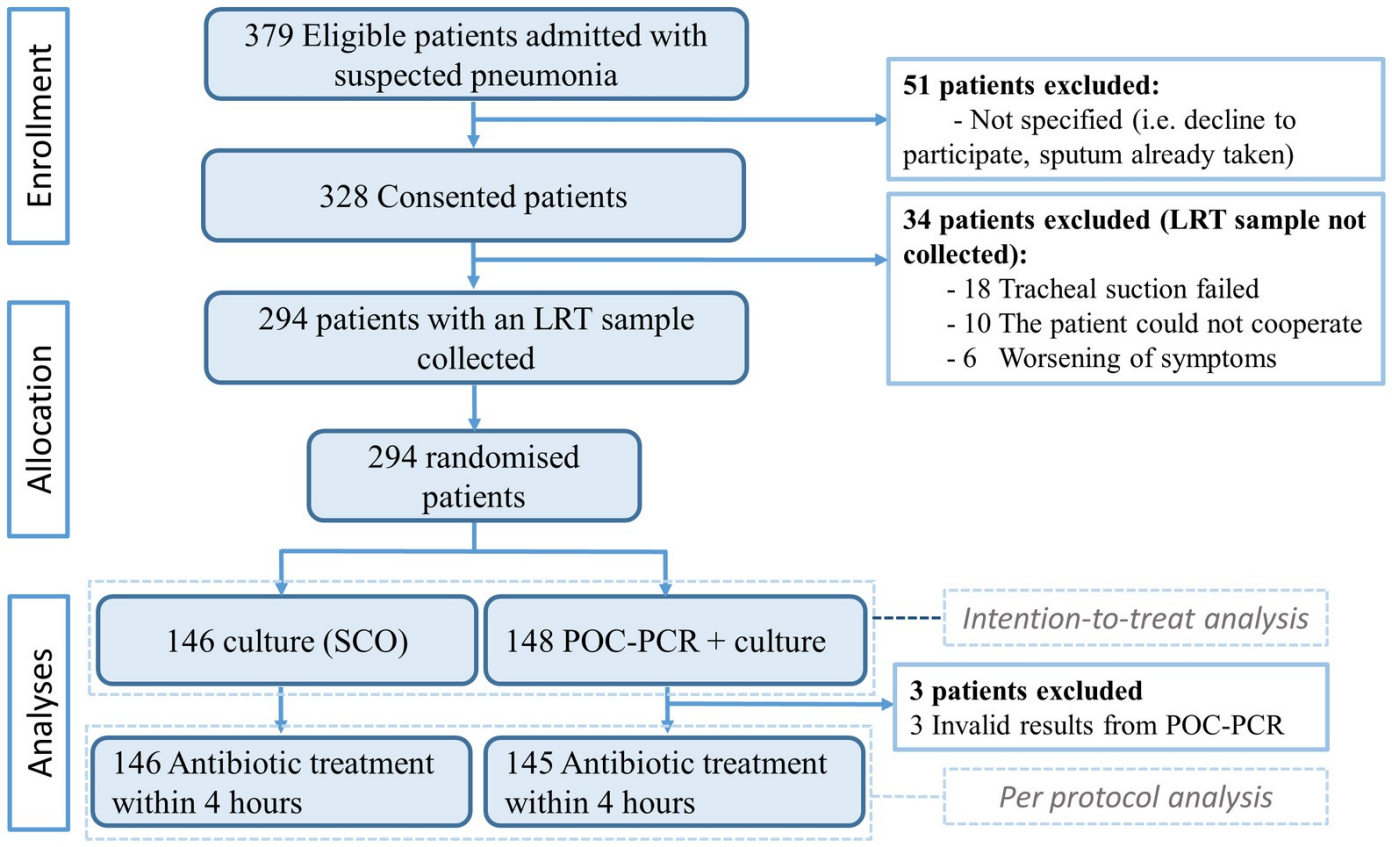
Trial profile. LRT, lower respiratory tract; POC-PCR, point-of-care polymerase chain reaction; SCO, standard care only.

Patients admitted with suspected CAP were enrolled from March 1, 2021 to February 28, 2022. The last follow-up for mortality and readmission was on April 1, 2022. We screened 379 patients for eligibility and collected 294 (77.6%) LRT samples (78.4% tracheal secretions and 21.6% expectorated sputa) from patients who underwent randomisation. The 294 patients were allocated to either the POC-PCR group (148 patients (50.4%)) or the SCO group (146 patients (49.6%)), and those patients were included in the intention-to-treat analysis. Per protocol analyses for the primary outcome included 291 (99.0%) patients with no or narrow antibiotic treatment registered within 4 h (POC-PCR 145 (49.8%) and SCO 146 (50.2%)) (Fig 1).

### Baseline data

Demographic and clinical characteristics are shown in Table 1.

**Table 1:** Baseline characteristics of patients included in the analysis.

Allocation	SCO *n* = 146	POC-PCR *n* = 145	Total *n* = 291
Age, median years (IQR)	72.5 (59.0; 81.0)	74.0 (61.5; 81.0)	73.0 (60.0; 81.0)
Gender (male), *n* (%)	70 (47.9)	78 (53.8)	148 (51.0)
Activities of daily living^a^, *n* (%)	45 (31.0)	33 (22.3)	78 (26.6)
Nursing home resident, *n* (%)	15 (10.3)	14 (9.5)	29 (9.9)
Patients with a confirmed CAP diagnosis^b^, *n* (%)	83 (56.8)	89 (61.4)	172 (59.1)
HRCT findings suggestive of pneumonia, *n* (%)	66 (45.2)	79 (54.5)	145 (49.8)
Type of respiratory samples, *n* (%)			
Tracheal secretions	112 (76.7)	116 (80.0)	228 (78.4)
Expectorated sputa	34 (23.3)	29 (20.0)	63 (21.6)
Blood culture, *n* (%)	127 (86.9)	120 (83.3)	247 (85.2)
Bloodstream infections	12 (8.2)	6 (4.1)	18 (6.2)
Urine culture, *n* (%)	124 (84.9)	119 (82.6)	243 (83.8)
Bacteriuria^c^	25 (20.2)	34 (28.3)	59 (24.2)
**SYMPTOMS**			
Cough *n* (%)	102 (71.3)	104 (72.2)	206 (71.8)
Expectoration, *n* (%)	85 (59.4)	78 (54.2)	163 (56.8)
Breast tightness, *n* (%)	44 (31.2)	46 (31.7)	90 (31.5)
Dyspnea, *n* (%)	104 (72.7)	108 (75.0)	212 (73.9)
**SEVERITY ASSESSMENT**			
CURB-65^d^ ≥3, *n* (%)	24 (16.4)	18 (12.4)	42 (14.4)
Glasgow Coma Scale <15, *n* (%)	7 (4.8)	6 (4.1)	13 (4.4)
Triage ≥2, *n* (%)	62 (42.5)	40 (27.6)	**102 (35.1)** †
**COMORBIDITIES**			
Chronic obstructive pulmonary disease, *n* (%)	42 (28.8)	51 (35.2)	93 (32.0)
Neurological disease, *n* (%)	27 (18.5)	28 (19.3)	55 (19.0)
Cardiovascular disease, *n* (%)	56 (38.4)	62 (42.8)	118 (40.5)
Endocrinological disease, *n* (%)	49 (33.6)	43 (29.7)	92 (31.6)
**VITAL PARAMETERS**			
Oxygen saturation, median (IQR)	94.0 (91.0; 96.0)	93.0 (92.0; 96.0)	94.0 (92.0; 96.0)
Respiratory frequency/min, median (IQR)	22.0 (20.0; 25.0)	20.0 (18.0; 24.0)	22.0 (18.0; 24.0)
Heart rate/min, mean (SD)	93.8 (18.2)	92.2 (17.6)	93.0 (17.9)
Systolic blood pressure mmHg, mean (SD)	134.7 (20.3)	135.4 (22.1)	135.0 (21.2)
Diastolic blood pressure mmHg, mean (SD)	75.2 (14.5)	76.0 (16.9)	75.6 (15.7)
Temperature °C, mean (SD)	37.6 (1.0)	37.5 (0.9)	37.6 (1.0)
**BLOOD TESTS**			
C-reactive protein mg/L, median (IQR)	86.5 (30.8; 170.8)	82.0 (30.5; 178.0)	82.0 (31.0; 174.0)
Leucocytes 10^9^/L, median (IQR)	11.1 (8.5; 15.6)	11.3 (8.5; 14.8)	11.2 (8.5; 15.2)
Neutrophils 10^9^/L, median (IQR)	8.2 (6.0; 13.1)	8.9 (6.2; 12.5)	8.7 (6.1; 12.6)
**ANTIBIOTIC TREATMENT and VACCINE STATUS**			
Antibiotic treatment before admission^f^, *n* (%)	38 (26.0)	36 (24.8)	74 (25.4)
Antibiotic treatment at admission, *n* (%)	32 (21.9)	30 (20.7)	62 (21.3)
Allergy to antibiotics, *n* (%)	9 (6.2)	12 (8.3)	21 (7.2)
Pneumococcal vaccine within 5 years, *n* (%)	75 (51.4)	84 (57.9)	159 (54.6)
Influenza vaccine (season 2020/2021), *n* (%)	103 (70.5)	105 (72.4)	208 (71.5)

Data are *n* (%): numbers (percentages), median (IQR: interquartile range), or mean (SD: standard deviation).

^a^Activities of daily living: One or more dependencies related to bathing, dressing, toileting, transfer, continence, and eating.

^b^The confirmed CAP diagnosis was assigned by an expert panel of experienced emergency and infectious disease experts in acute infections based on all clinical information from the medical record within the first week of ED admission, including a chest computed tomography.

^c^Bacteriuria >10^4 bacteria/mL (E*nterobacteriaceae)* or >10^5 (others).

^d^CURB-65: confusion, blood urea nitrogen >7 mmol/l, respiratory rate ≥30 breaths per minute, blood pressure <90 mmHg systolic or ≤60 mmHg diastolic, age ≥65 years.

^e^Triage: Danish emergency process triage [34].

^f^Antibiotic treatment within 1 month prior to admission.

^†^*p* = 0.001

CAP, community-acquired pneumonia; ED, emergency department; HRCT, high-resolution computed tomography; mmHg, millimetre(s) of mercury; mg/L, milligrammes per litre; POC-PCR, point-of-care polymerase chain reaction; SCO standard care only.

Number of patients prescribed “no or narrow,” targeted, and adequate antibiotic at 4 h, 48 h, and 5 days is presented in Table 2. Because of the observed difference in triage between the intervention and control, unadjusted and adjusted results are presented in Tables 3 and 4.

**Table 2:** Absolute values for “no or narrow (no and narrow), targeted and adequate treatments” at 4 h, 48 h, and day 5. Analyses of targeted and adequate treatment were based on 55 positive culture results from 290 patients.

**Patients with prescriptions of “no or narrow” antibiotics**									
**Timeline**	**4 hours, *n* = 291**			**48 hours, *n* = 291**			**5th day, *n* = 290**		
	POC-PCR^1^ 145 (49.8%)	SCO^2^ 146 (50.2%)	Total 291 (100%)	POC-PCR^1^ 145 (49.8%)	SCO^2^ 146 (50.2%)	Total 291 (100%)	POC-PCR^1^ 144 (49.7%)	SCO^2^ 146 (50.3%)	Total 290 (99.7%)
**No or Narrow antibiotic**	91 (62.8%)	87 (59.6%)	178 (61.2%)	88 (60.7%)	90 (61.6%)	178 (61.2%)	88 (61.1%)	95 (65.1%)	183 (63.1%)
**-No antibiotic**	30 (20.7%)	29 (19.9%)	59 (20.3%)	31 (21.4%)	28 (19.2%)	59 (20.3%)	33 (22.9%)	36 (24.7%)	69 (23.8%)
**-Narrow antibiotic**	61 (42.1%)	58 (39.7%)	119 (40.9%)	57 (39.3%)	62 (42.4%)	119 (40.9%)	55 (38.2%)	59 (40.4%)	114 (39.3%)
**Patients with positive culture results**									
**Timeline**	**4 hours, *n* = 55**			**48 hours, *n* = 55**			**5th day, *n* = 55**		
	POC-PCR^1^ 26 (47%)	SCO^2^ 29 (53%)	Total 55 (100%)	POC-PCR^1^ 26 (47%)	SCO^2^ 29 (53%)	Total 55 (100%)	POC-PCR^1^ 26 (47%)	SCO^2^ 29 (53%)	Total 55 (100%)
**Target antibiotic**	15 (57.7%)	7 (24.1%)	22 (40.0%)	17 (65.4%)	10 (34.5%)	27 (49.1%)	14 (53.9%)	15 (51.7%)	29 (52.7%)
**Adequate antibiotic**	19 (73.1%)	17 (58.6%)	36 (65.5%)	20 (76.9%)	18 (62.1%)	38 (69.1%)	19 (73.1%)	19 (65.5%)	38 (69.1%)

^1^POC-PCR in addition to routine culture.

^2^SCO.

POC-PCR, point-of-care polymerase chain reaction; SCO, standard care only.

**Table 3:** Unadjusted and adjusted per protocol analyses for the primary and secondary outcomes: Prescriptions of no or narrow, targeted, and adequate antibiotic treatment at 4 h, 48 h, and day 5. The control group (SCO) is the reference. Analyses of targeted and adequate treatment were based on 55 positive culture results and routine PCR from 290 patients.

Timeline	4 hours (*n* = 291)		48 hours (*n* = 291)		5 days (*n* = 290)	
	OR (95% CI)	*p*-Value	OR (95% CI)	*p*-Value	OR (95% CI)	*p*-Value
**Primary outcome**						
No or narrow antibiotic	1.14 (0.97; 1.34)	0.101	-	-	-	-
Adjusted for triage	1.05 (0.73; 1.51)	0.772				
**Secondary outcomes**						
No or narrow antibiotic	-	-	0.96 (0.87; 1.04)	0.373	0.84 (0.73; 0.97)	0.021
Adjusted for triage	-	-	0.91 (0.82; 1.00)	0.065	0.81 (0.72; 0.91)	0.001
**Timeline**	**4 hours (*n* = 55)**		**48 hours (*n* = 55)**		**5 days (*n* = 55)**	
**Secondary outcomes**						
Target antibiotic	4.28 (2.51; 7.32)	<0.001	3.58 (1.39; 9.26)	0.008	1.09 (0.65; 1.83)	0.749
Adjusted for triage	5.68 (2.49; 12.94)	<0.001	4.20 (1.87; 9.40)	<0.001	1.08 (0.61; 1.91)	0.786
Adequate antibiotic	1.91 (0.68; 5.40)	0.219	2.04 (1.32; 3.14)	0.001	1.43 (1.33; 1.54)	<0.001
Adjusted for triage	2.11 (0.56; 7.96)	0.267	2.11 (1.23; 3.61)	0.006	1.40 (1.18; 1.66)	<0.001

CI, confidence interval; OR, odds ratio; PCR, polymerase chain reaction; SCO, standard care only.

**Table 4:** Adverse events and LOS for 291 patients.

Adverse events	SCO	POC-PCR	OR (95% CI) *p*-value	OR (95% CI) *p*-value
	Event (*n* = 146)	Event (*n* = 145)	Crude	Adjusted for triage
30 Days mortality^1^	4	5	1.26 (0.33; 4.81) 0.728	1.24 (0.32; 4.82) 0.749
In-hospital mortality^2^	3	3	1.00 (0.19; 5.07) 0.993	0.98 (0.19; 5.06) 0.986
Admission to ICU^3^	5	2	0.39 (0.07; 2.06) 0.271	0.54 (0.10; 2.91) 0.475
Readmission to hospital^4^	20	17	0.83 (0.41; 1.67) 0.614	0.90 (0.43; 1.86) 0.787
Adverse events in total^5^	32	27	0.96 (0.51; 1.77) 0.896	1.04 (0.55; 1.97) 0.899
	Days	Days	**IRR (95% CI) *p*-value**	
LOS^6^ (average in days)	5.2	4.2	0.80 (0.62; 1.04), 0.098	
Adjusted for triage	4.3	3.6	0.82 (0.63; 1.07), 0.164	

^1^Mortality within 30 days from admission to the ED.

^2^Patient mortality during the current hospitalisation.

^3^Transfer to ICU during the current hospitalisation.

^4^Admission within a 30-day period after discharge from current admission.

^5^Total of numbers of adverse events per patient.

^6^Defined as the time (in days) spent in hospital during the current admission (days from admission to hospital discharge).

CI, confidence interval; ED, emergency department; ICU, intensive care unit; LOS, length of stay; OR, odds ratio; POC-PCR, point-of-care polymerase chain reaction; SCO, standard care only.

### Prescription of no or narrow-spectrum antibiotics

There were 3 missing samples due to POC-PCR assay failure. Thus, no clinical characteristics influenced the missing mechanism. Therefore, we believe the data are missing completely at random. However, for sensitivity reasons, multiple imputation was performed. Results from per protocol and intention-to-treat analysis were similar. POC-PCR was not superior to SCO regarding prescriptions of no or narrow-spectrum antibiotics within 4 h after admission. Intention-to-treat analyses of 294 patients yielded an OR 1.13; (95% CI [0.96, 1.34] *p* = 0.134), and per protocol analysis of 291 patients resulted in an OR 1.14; (95% CI [0.97, 1.34] *p* = 0.101). We found a statistically significant difference on day 5 but not 48 h after admission (Table 3).

### Prescription of targeted and adequate antibiotics

**Fig 2:**
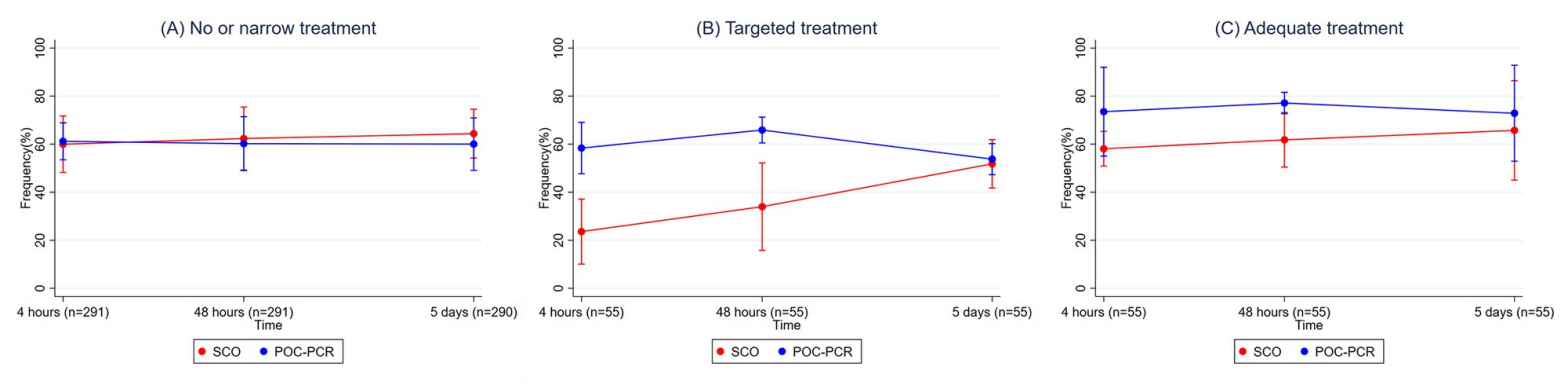
Changes in (2A) no or narrow-, (2B) targeted-, and (2C) adequate treatment prescription at 4 h, 48 h, and day 5. Targeted and adequate treatments are based on culture results and routine PCR and include a sample of 55 patients. Results were adjusted for triage. POC-PCR, point-of-care polymerase chain reaction; SCO, standard care only.

Prespecified analysis of targeted antibiotic treatment and exploratory analyses of adequate antibiotics were based on positive culture results from 290 specimens after exclusion of one sample missing from the culture analysis. We identified 68 (23%) bacterial agents from 55 (19%) patients. Targeted treatment was used significantly more often in the POC-PCR compared with the SCO group at both 4 h and 48 h but not at day 5 (Table 3). Analysis of adequate treatment did not show a statistically significant difference between the groups at 4 h but more patients were treated with adequate antibiotics at 48 h and on day 5 in the POC-PCR compared to the SCO group (Table 3). A graphical presentation of changes in (2A) no or narrow, (2B) targeted, and (2C) adequate treatment for both groups is presented in Fig 2.

### Adverse events

There were no statistically significant differences between POC-PCR and SCO regarding patient 30-day mortality, in-hospital mortality, admission to ICU, 30-day readmission, and LOS (Table 4).

## Discussion

In this randomised study, adding sputum-POC-PCR to our diagnostic setup did not affect prescriptions of no or narrow-spectrum antibiotics during the first 2 days of admission, but less patients in the POC-PCR-group were treated with no or narrow-spectrum antibiotics after 5 days. Interestingly, patients in the POC-PCR-group were more likely to receive early targeted and adequate treatment. Number of readmissions, ICU admissions, and mortality were unchanged but we found a nonsignificant one-day reduction in LOS. Several prospective studies have reported sputum-POC-PCR as a method to support clinical decisions by fast and accurate detection of CAP pathogens [21–23,35]. Studies have shown a reduction in both use of intravenous antibiotics and number of days treated with antibiotics. In contrast to our study, most previous studies in ED settings only used panels for detecting upper respiratory pathogens [25,26,36,37].

Our outcomes were different focusing on type of antibiotic instead of length of treatment and route of administration, but nevertheless, the failure of POC-PCR to increase the use of no or narrow-spectrum antibiotics may seem to contrast these previous results.

There are some likely explanations. In an international context, the level of antimicrobial resistance is very low in Denmark and most pneumococci and *H*. *influenzae* are susceptible to penicillins [10]. Consequently, Danish treatment guidelines recommend relatively narrow-spectrum penicillins for CAP and reserve broad-spectrum antibiotics for severe pneumonia and/or sepsis [11,12]. This may have affected the study. For example, a patient with severe CAP may have been treated with penicillin instead of broad-spectrum antibiotics if POC-PCR detected pneumococci and another patient with mild CAP may have been treated with broad-spectrum antibiotics instead of penicillin if POC-PCR detected *M*. *catarrhalis*. Both actions were in agreement with the provided action card and both actions would result in a more targeted treatment—but also blur the effect of POC-PCR. This explanation is in line with the observation that patients in the POC-PCR group were more likely to receive early targeted and adequate treatment. In addition, the detection of *Enterobacterales* and *Pseudomonas aeruginosa* with POC-PCR may result in broad antimicrobial therapy even though they rarely cause CAP in a medical ED [38]. We excluded *Enterobacterales* from the analysis of targeted and adequate treatment due to the low incidence (1.3%) and because they usually represent colonisation [38].

Another possible explanation is the very low prevalence of common respiratory viruses in the study period related to the SARS-CoV-2 pandemic [39]. In other studies, virus accounted for 20% to 40% of CAP cases [19,20,37]. Some patients with CAP and a detected viral cause may be treated without antibiotics, and it is therefore possible that POC-PCR would have reduced the use of antibiotics in a period with a higher transmission of respiratory viruses.

The increased prescription of targeted and adequate antibiotics in the POC-PCR-group within the first 2 days is an interesting observation. It is based on analysis of a small subset of culture-positive samples; therefore, it is unknown if the result completely or in part can be extrapolated to the rest of the study population. Nevertheless, it highlights the question if POC-PCR improves patient outcome. We did not find any difference in mortality or transferal to ICU—but the number of events was very low. There was no difference in the number of readmissions but we did find a nonsignificant reduction in LOS from 4.3 to 3.6 days (*p* = 0.164) when adjusted for triage. It was not significant, but it might on the other hand reflect improved patient treatment and a possible reduction in LOS of almost 20% for one of the most common infections in the ED is very interesting from a hospital management and economic perspective.

At day 5, more patients in the SCO group were treated with no or narrow-spectrum antibiotics and there was no difference in the use of targeted antibiotics. This observation may be explained by routine microbiological results being available between day 2 and 5—allowing adjustment of treatment. Even though, we detected statistically significant differences they might be without clinical significance as they were quite small and day 5 is at the end of our recommended treatment duration. The strength of our study is the pragmatic multicentre, RCT design. The randomised design ensured that severity of illness, CAP diagnosis, and other patient characteristics were distributed equally between intervention and control group, and therefore causal inference is likely as the assumption of positivity is fulfilled. The POC-PCR analysis was integrated in the usual workflow in our ED suggesting that the test is technically feasible and easy to implement in other EDs. Project assistants were trained in collecting LRT-specimens and in using the POC-PCR platform and the primary investigator monitored the project closely to ensure a high level of internal validity. Almost 80% of the collected samples were tracheal secretions and this may have increased the reliability of the microbiological results by reducing upper airway contamination [40,41]. To ensure a uniform and correct clinical interpretation, we provided all POC-PCR results with a clear guideline-based action card.

There are also a number of limitations. Only few patients with CURB-65 scores ≥3 (14.4%) were included in the study. The inability to consent is likely linked to severe disease and acute cognitive impairment. In addition, restriction to weekdays and daytime may have reduced the number of severe cases as admission on weekends and at night are known to be associated with increased mortality and risk of referral to ICU [42]. Therefore, results can only be generalised to patients admitted on weekdays during daytime. In the secondary analysis of targeted and adequate treatment, only few culture-positive samples were included. The sensitivity of culture may be very low, and a high number of patients were treated with antibiotics before admission [43]. We could have circumvented this challenge by also analysing samples in the SCO group with FilmArray with a random disclosure design where results only are available in the intervention group. It would also allow subgroup analysis to investigate the effect of POC-PCR separately in test-positive and test-negative patients. It would straighten the results, leading to evidence-practice recommendations for implementing the test in clinical practice. However, it would be more expensive and may introduce ethical issues [44,45].

Both culture and POC-PCR may detect commensals, which was stated clearly in the provided action card. It is therefore possible that the clinicians in some situations chose to ignore the result—e.g., based on severity of illness, response to current treatment, fear of prescribing inadequate treatment, likelihood of commensal pathogen, and expected virulence of the pathogen [46]. We did not measure to what extent the action card recommendations were followed.

A possible interpretation of the overall results is that the current restrictive prescribing strategy in Denmark may be unable to provide targeted and adequate treatment for some patients. This may be overcome by introducing broad-spectrum empirical regimes—but that would fuel a further rise in resistance, may introduce side effects, and go against our general antimicrobial stewardship interventions. However, as indicated in this study, we might get around this problem by introducing fast and sensitive diagnostic methods. Future studies should focus on (i) the impact of POC-PCR on clinical outcome in a larger scale—e.g., LOS, length of treatment, and patient quality of life; (ii) hospitalisation costs; and (iii) the use of adequate and target treatment in a blinded setup where sensitive molecular methods are applied in both intervention and control groups. In conclusion, in this randomised trial introduction of POC-PCR did not increase the proportion of patients prescribed no or narrow-spectrum antibiotics but it might increase early treatment with adequate and targeted antibiotics and may be associated with a reduced LOS. The results apply to a setting with restrictive use of antibiotics and a very low level of antimicrobial resistance and may be quite different in other settings. Fast and accurate diagnostic tools may aid to maintain a restrictive use of antibiotics in the future.

## Supporting information

S1 Table Empirical treatment guidelines of CAP of the region of Southern Denmark. (PDF)

S2 Table Standard care procedures in our emergency departments. (PDF)

S3 Table Targets of the Biofire FilmArray Pneumonia Panel plus. (PDF)

S4 Table Classification of “Narrow antibiotic” treatment. (PDF)

S5 Table Classification of “targeted and adequate” treatment. (PDF)

S1 Text CONSORT checklist. (PDF)

S2 Text Trial protocol. (PDF)

S3 Text Written consent and information form. (PDF)

S4 Text Action card. (PDF)

S5 Text Data availability and data sharing plan. (PDF)

## Abbreviations

CAP: community-acquired pneumonia
CI: confidence interval
COVID-19: Coronavirus Disease 2019
ED: emergency department
ICU: intensive care unit
LOS: length of stay
LRT: lower respiratory tract
OR: odds ratio
PCR: polymerase chain reaction
POC: point-of-care
RCT: randomised controlled trial
SCO: standard care only
