# Supplementary material for: Evaluation of point-of-care multiplex polymerase chain reaction in guiding antibiotic treatment of patients acutely admitted with suspected community-acquired pneumonia in Denmark: A multicentre randomised controlled trial
Source: PLoS Med. 2023 Nov 28;20(11):e1004314. doi: 10.1371/journal.pmed.1004314 (PMC10684013; doi:10.1371/journal.pmed.1004314)
Supplement: S1 Table — (PDF) [file pmed.1004314.s001.pdf]

Table S1: Empirical treatment guidelines of CAP of the region of Southern Denmark

Severity of CAP	First choice	Penicillin allergy	Therapy duration (iv* and oral)
CURB-65: 0-2	Benzylpenicillin 1.2g (2 mill.IE) x4 iv. or Phenoxymethylpenicillin 0.6g (1 mill.IE) x 4 oral	Cefuroxime 1.5g x 3 iv. or Roxithromycin 300mg x1 oral	5 days
CURB-65 ≥ 3	Benzylpenicillin 1.2g (2 mill.IE) x 4 iv. + Azithromycin† 500mg x 1 iv.	Cefuroxime 1.5g x 3 iv. + Azithromycin 500mg x 1 iv.	7 days
CURB-65 ≥ 3+□	Piperacillin-tazobactam 4/0.5gx3 iv. + Azithromycin 500mg x1 iv.	Cefuroxime 1.5g x 3 iv. + Azithromycin 500mg x 1 iv.	7 days

*Intravenous route. † Azithromycin: The treatment is extended only if PCR is positive for *Legionella pneumophila*, *Mycoplasma pneumoniae*, or *Chlamydophila pneumoniae*. Azithromycin (500 mg iv.) has approx. 2-4 days therapeutic coverage. □CURB-65 ≥ 3+: Confusion, urea, respiratory frequency, blood pressure + radiological involvement of multiple lung lobes, or hypoxia with O_2 saturation < 92%, or sepsis.
