# Supplementary material for: Evaluation of point-of-care multiplex polymerase chain reaction in guiding antibiotic treatment of patients acutely admitted with suspected community-acquired pneumonia in Denmark: A multicentre randomised controlled trial
Source: PLoS Med. 2023 Nov 28;20(11):e1004314. doi: 10.1371/journal.pmed.1004314 (PMC10684013; doi:10.1371/journal.pmed.1004314)
Supplement: S2 Table — (PDF) [file pmed.1004314.s002.pdf]

Table S2: Standard care procedures in our emergency departments

TIMEPOINT	30 min.	Within 1-hour	Within 4-hours	48-hours	Day 5
Clinical assessment	x				
Tracheal secretions collection		x			
Microbiological results				x	
Blood samples collection		x			
Biochemistry results			x		
Chest X-ray			x		
Empirical treatment			x		
Adjustment of therapy				x	x
