# Supplementary material for: Evaluation of point-of-care multiplex polymerase chain reaction in guiding antibiotic treatment of patients acutely admitted with suspected community-acquired pneumonia in Denmark: A multicentre randomised controlled trial
Source: PLoS Med. 2023 Nov 28;20(11):e1004314. doi: 10.1371/journal.pmed.1004314 (PMC10684013; doi:10.1371/journal.pmed.1004314)
Supplement: S3 Table — (PDF) [file pmed.1004314.s003.pdf]

Table S3: Targets of the Biofire FilmArray Pneumonia Panel plus (Biomérieux, Marcy l'Etoile, France)

Bacteria	Atypical bacteria	Antimicrobial resistance genes
Acinetobacter calcoaceticus baumannii complex	*Chlamydophilia pneumoniae*	*mecA/C and MREJ*
Enterobacter cloacae complex	*Legionella pneumophila*	*KPC,*
Escherichia coli	*Mycoplasma pneumonia*	*CTX-M*
Haemophilus influenzae		*NDM*
Klebsiella aerogenes		*Oxa48-like*
Klebsiella oxytoca	**Viruses**	*VIM*
Klebsiella pneumoniae group	*Influenza A*	*IMP*
Moraxella catarrhalis	*Influenza B*	
Proteus spp.	*Adenovirus**	
Pseudomonas aeruginosa	*Parainfluenza virus*	
Serratia marcescens	*Coronavirus (CoV)***	
Staphylococcus aureus	*Human metapneumovirus*	
Streptococcus agalactiae,	*Human rhinovirus/enterovirus*	
Streptococcus pneumoniae	*MERS-CoV*	
Streptococcus pyogenes	*Respiratory syncytial virus*	

* Adenovirus is not included in our analysis due to the expiration date specific for Adenovirus announced by Biofire Nordic 21. July 2021. Biofire FilmArray Pneumonia plus (PN plus) Panel (RFIT-ASY-0142 and RFIT-ASY-0143).
** Coronavirus (CoV): serological variants (229E, OC43, HKU1, NL63)
