# Supplementary material for: Evaluation of point-of-care multiplex polymerase chain reaction in guiding antibiotic treatment of patients acutely admitted with suspected community-acquired pneumonia in Denmark: A multicentre randomised controlled trial
Source: PLoS Med. 2023 Nov 28;20(11):e1004314. doi: 10.1371/journal.pmed.1004314 (PMC10684013; doi:10.1371/journal.pmed.1004314)
Supplement: S4 Table — (PDF) [file pmed.1004314.s004.pdf]

Table S4: Classification of ”Narrow antibiotic” treatment

Classification	Antibiotic	CAVE
**Narrow-spectrum antibiotics against CAP and**	Benzylpenicillin<br>Phenoxymethylpenicillin<br>Ampicillin<br>Pivampicillin<br>Amoxicillin	No
	Macrolides<br>Cefuroxime	Yes
**No antibiotics**	-	-
**Broad-spectrum antibiotics and antibiotics not directed against CAP**	*Monotherapy:*<br>Amoxicillin/Clavulanic acid’<br>Piperacillin/Tazobactam<br>Doxycycline<br>Tetracycline<br>Moxifloxacin<br>Sulfamethoxazole and trimethoprim<br>Macrolides	No
	Cefuroxime	Is considered narrow in case of CAVE
	Combination therapy:<br>Benzylpenicillin *or*<br>Phenoxymethylpenicillin *or*<br>Ampicillin *or*<br>Pivampicillin *or*<br>Amoxicillin *or*<br>Amoxicillin/Clavulanic acid’*or*<br>Tazobactam/Piperacillin *or*<br>Cefuroxim<br><br>*Combined with:*<br>Doxycylin *or*<br>Tetracyclin *or*<br>Ciprofloxacin *or*<br>Moxifloxacin *or*<br>Macrolides<br><br>*Antibiotics not directed against CAP:*<br>Dicloxacillin<br>Cloxacillin<br>Flucloxacillin<br>Pivmecillinam<br>Mecillinam	No

	Tigecyclin<br>Cefalexin<br>Cefazolin<br>Cefotaxim<br>Ceftazidim<br>Ceftriaxon<br>Cefepime<br>Ceftolozan/Tazobactam<br>Ceftazidim/Avibactam<br>Meropenem<br>Ertapenem<br>Imipenem- cilastatin<br>Trimethoprim<br>Sulfamethizol<br>Tobramycin<br>Gentamicin<br>Clindamycin<br>Ciprofloxacin	

Macrolides* = Erythromycin or roxithromycin or clarithromycin or azithromycin
