# Supplementary material for: Evaluation of point-of-care multiplex polymerase chain reaction in guiding antibiotic treatment of patients acutely admitted with suspected community-acquired pneumonia in Denmark: A multicentre randomised controlled trial
Source: PLoS Med. 2023 Nov 28;20(11):e1004314. doi: 10.1371/journal.pmed.1004314 (PMC10684013; doi:10.1371/journal.pmed.1004314)
Supplement: S5 Table — (PDF) [file pmed.1004314.s005.pdf]

Table S5: Classification of ”targeted and adequate” treatment

Antimicrobial	Microbiological agents						
	S. pneumoniae	H.influenzae	M.catarrhalis	P.aeruginosa	S. aureus	Hemolytic streptococcus	L. pneumophila
Benzylpenicillin	Targeted	Targeted	not relevant	not relevant	not relevant	Targeted	not relevant
Phenoxymethyl-penicillin	Targeted	not relevant	not relevant	not relevant	not relevant	Targeted	not relevant
Ampicillin	Adequate	Targeted	not relevant	not relevant	not relevant	Adequate	not relevant
Pivampicillin	Adequate	Targeted	not relevant	not relevant	not relevant	Adequate	not relevant
Amoxicillin	Adequate	Targeted	not relevant	not relevant	not relevant	Adequate	not relevant
Pivmecillinam	not relevant	not relevant	not relevant	not relevant	not relevant	not relevant	not relevant
Mecillinam	not relevant	not relevant	not relevant	not relevant	not relevant	not relevant	not relevant
Dicloxacillin	not relevant	not relevant	not relevant	not relevant	Targeted	not relevant	not relevant
Cloxacillin	not relevant	not relevant	not relevant	not relevant	Targeted	not relevant	not relevant
Flucloxacillin	not relevant	not relevant	not relevant	not relevant	Targeted	not relevant	not relevant
Amoxicillin/ Clavulanic acid’	Adequate	Targeted	Targeted	not relevant	Adequate	Adequate	not relevant
Tazobactam/ Piperacillin	Adequate	Targeted	Targeted	Targeted	Adequate	Adequate	not relevant
Cefuroxime	CAVE/Targeted	CAVE/Targeted	Targeted	not relevant	CAVE/Targeted	CAVE/Targeted	not relevant
Cefotaxim	Adequate	Adequate	Adequate	not relevant	Adequate	Adequate	not relevant
Ceftriaxon	Adequate	Adequate	Adequate	not relevant	Adequate	Adequate	not relevant
Ceftazidim	not relevant	Adequate	Adequate	Targeted	not relevant	Adequate	not relevant
Cefepime	Adequate	Adequate	Adequate	Adequate	Adequate	Adequate	not relevant
Meropenem	Adequate	Adequate	Adequate	Adequate	Adequate	Adequate	not relevant
Ertapenem	Adequate	Adequate	Adequate	not relevant	Adequate	Adequate	not relevant
Imipenem and cilastatin	Adequate	Adequate	Adequate	Adequate	Adequate	Adequate	not relevant
Macrolides*	CAVE/Targeted	not relevant	Targeted	not relevant	not relevant	CAVE/Targeted	Targeted
Clindamycin	CAVE/Targeted	not relevant	not relevant	not relevant	CAVE/Targeted	CAVE/Targeted	not relevant
Doxycylin	Adequate	CAVE/Targeted	Adequate	not relevant	Adequate	Adequate	Targeted
Tetracyclin	Adequate	CAVE/Targeted	Adequate	not relevant	Adequate	Adequate	Targeted
Tigecyclin	Adequate	Adequate	Adequate	not relevant	Adequate	Adequate	not relevant
Tobramycin	not relevant	not relevant	not relevant	Targeted	not relevant	not relevant	not relevant
Gentamicin	not relevant	not relevant	not relevant	Targeted	not relevant	not relevant	not relevant
Ciprofloxacin	not relevant	CAVE/Targeted	Adequate	Targeted	not relevant	not relevant	Targeted
Moxifloxacin	Adequate	Adequate	Adequate	not relevant	Adequate	Adequate	Targeted
Trimethoprim	not relevant	not relevant	not relevant	not relevant	not relevant	not relevant	not relevant
Sulfamethizol	not relevant	not relevant	not relevant	not relevant	not relevant	not relevant	not relevant
Sulfamethoxazole and trimethoprim	Adequate	Adequate	Adequate	not relevant	Adequate	Adequate	not relevant

Green (targeted treatment): Antibiotics directed against a bacterial pathogen detected by culture without being unnecessary board-spectrum.
Blue (CAVE/Targeted): Considered targeted treatment if the patient was registered as allergic to penicillins.
Yellow (Adequate): Antibiotics that are active against the bacterial pathogen detected by culture.
Orange (Not relevant): Antibiotics that are not recommended and/or regarded inactive against the bacterial pathogen detected by culture.
*Macrolides: Erythromycin or roxithromycin or clarithromycin or azithromycin.
