# Supplementary material for: Evaluation of point-of-care multiplex polymerase chain reaction in guiding antibiotic treatment of patients acutely admitted with suspected community-acquired pneumonia in Denmark: A multicentre randomised controlled trial
Source: PLoS Med. 2023 Nov 28;20(11):e1004314. doi: 10.1371/journal.pmed.1004314 (PMC10684013; doi:10.1371/journal.pmed.1004314)
Supplement: S1 Text — (PDF) [file pmed.1004314.s006.pdf]

CONSORT 2010 checklist of information to include when reporting a randomised trial

Section/Topic	Item No	Checklist item	Reported section (§) and paragraph (¶) number
**Title and abstract**			
	1a	Identification as a randomised trial in the title	Title
	1b	Structured summary of trial design, methods, results, and conclusions (for specific guidance see CONSORT for abstracts)	§ Abstract
**Introduction**			
Background and objectives	2a	Scientific background and explanation of rationale	§ Introduction
	2b	Specific objectives or hypotheses	§ Introduction ¶ 5
**Methods**			
Trial design	3a	Description of trial design (such as parallel, factorial) including allocation ratio	§ Trial design ¶ 1<br>§ Randomisation and masking ¶ 1
	3b	Important changes to methods after trial commencement (such as eligibility criteria), with reasons	n/a
Participants	4a	Eligibility criteria for participants	§ Participants ¶ 1
	4b	Settings and locations where the data were collected	§ Setting ¶ 1
Interventions	5	The interventions for each group with sufficient details to allow replication, including how and when they were actually administered	§ Intervention
Outcomes	6a	Completely defined pre-specified primary and secondary outcome measures, including how and when they were assessed	§ Outcomes
	6b	Any changes to trial outcomes after the trial commenced, with reasons	§ Outcomes ¶ 1
Sample size	7a	How sample size was determined	§ Statistical methods ¶ 1
	7b	When applicable, explanation of any interim analyses and stopping guidelines	n/a
Randomisation:			
Sequence generation	8a	Method used to generate the random allocation sequence	§ Randomisation and masking ¶ 1
	8b	Type of randomisation; details of any restriction (such as blocking and block size)	§ Randomisation and masking ¶ 1
Allocation concealment mechanism	9	Mechanism used to implement the random allocation sequence (such as sequentially numbered containers), describing any steps taken to conceal the sequence until interventions were assigned	§ Randomisation and masking ¶ 1
Implementation	10	Who generated the random allocation sequence, who enrolled participants, and who	§ Randomisation and masking ¶ 1

		assigned participants to interventions	
Blinding	11a	If done, who was blinded after assignment to interventions (for example, participants, care providers, those assessing outcomes) and how	§ Randomisation and masking ¶ 1
	11b	If relevant, description of the similarity of interventions	n/a
Statistical methods	12a	Statistical methods used to compare groups for primary and secondary outcomes	§ Statistical methods ¶ 3
	12b	Methods for additional analyses, such as subgroup analyses and adjusted analyses	§ Statistical methods ¶ 3
**Results**			
Participant flow (a diagram is strongly recommended)	13a	For each group, the numbers of participants who were randomly assigned, received intended treatment, and were analysed for the primary outcome	§ Results ¶ 1
	13b	For each group, losses and exclusions after randomisation, together with reasons	§ Prescription of no or narrow-spectrum antibiotics ¶ 1
Recruitment	14a	Dates defining the periods of recruitment and follow-up	§ Results ¶ 1
	14b	Why the trial ended or was stopped	n/a
Baseline data	15	A table showing baseline demographic and clinical characteristics for each group	§ Baseline data, Table 1
Numbers analysed	16	For each group, number of participants (denominator) included in each analysis and whether the analysis was by original assigned groups	§ Results ¶ 1
Outcomes and estimation	17a	For each primary and secondary outcome, results for each group, and the estimated effect size and its precision (such as 95% confidence interval)	§ Results Tables 2 + 3 and Figure 2
	17b	For binary outcomes, presentation of both absolute and relative effect sizes is recommended	§ Results Table 2
Ancillary analyses	18	Results of any other analyses performed, including subgroup analyses and adjusted analyses, distinguishing pre-specified from exploratory	§ Results Tables 3 + 4
Harms	19	All important harms or unintended effects in each group (for specific guidance see CONSORT for harms)	§ Results Tables 3 + 4
**Discussion**			
Limitations	20	Trial limitations, addressing sources of potential bias, imprecision, and, if relevant, multiplicity of analyses	§ Discussion ¶ 10 + 13
Generalisability	21	Generalisability (external validity, applicability) of the trial findings	§ Discussion ¶ 10 + 12
Interpretation	22	Interpretation consistent with results, balancing benefits and harms, and considering other relevant evidence	§ Discussion ¶ 10 + 12
**Other information**			
Registration	23	Registration number and name of trial registry	§ Trial design ¶ 2

Protocol	24	Where the full trial protocol can be accessed, if available	§ Trial design ¶ 2
Funding	25	Sources of funding and other support (such as supply of drugs), role of funders	§ Funding
