# Supplementary material for: Evaluation of point-of-care multiplex polymerase chain reaction in guiding antibiotic treatment of patients acutely admitted with suspected community-acquired pneumonia in Denmark: A multicentre randomised controlled trial
Source: PLoS Med. 2023 Nov 28;20(11):e1004314. doi: 10.1371/journal.pmed.1004314 (PMC10684013; doi:10.1371/journal.pmed.1004314)
Supplement: S2 Text — (PDF) [file pmed.1004314.s007.pdf]

Infectious Diseases in Emergency Departments (INDEED study)

– a protocol of a multifaceted diagnostic study

Content

Background 2
Antibiotic resistance 2
Urgent infections and diagnostic tools 2
Hypothesis, aim and objectives 3
Method 4
Study design 4
Setting 5
Population and eligibility criteria 6
Recruitment 6
Procedure 7
Outcomes and statistical analysis 11
Ethical considerations and risks 14
Research group 15
Significance of the study 16
Funding 16
Literature references 17

Background

Antibiotic resistance

Multi-resistant bacteria (MRB) is one of the major threats to the public health(1). In Denmark, the incidence of MRB is increasing (2) and every 20th patient in the Emergency Department (ED) in Denmark is a carrier of MRB(3). Denmark has focused on this challenge (4) by screening special groups for MRB (5, 6), and initiating campaigns to reduce antibiotic consumption mainly by reducing the use of broad-spectrum antibiotics in hospitals (4, 7).

The Danish Ministry of Health has made extensive efforts targeting the use of antibiotics in hospitals by establishing learning and quality teams (LQT). The experience from LQT identified the major obstacle in reducing the prescription of broad-spectrum antibiotics was insufficient knowledge concerning *if* the patient had a bacterial infection, *where* the infection was located and which kind of bacteria were involved in the infection.(8) Uncertainty in the answer to these three questions often leads a clinician to choose a broad-spectrum antibiotic at the onset of treatment. Unfortunately, often the prescription of a broad-spectrum antibiotic is rarely revised when laboratory results are available, mainly because the patient has often already been discharged(9).

Acute infections and diagnostic tools

The most common conditions among ED patients with suspected infection are community acquired pneumonia and acute pyelonephritis (defined in this study as an infection above the bladder i.e. pyelitis, nefritis, renal abscess and pyonephrosis) (10, 11). Pneumonia and acute pyelonephritis are urgent infections, and antibiotic treatment should be initiated within a few hours to avoid serious complications such as bacteremia, sepsis, organ failure, septic shock and death (12). A prerequisite for the appropriate use of antibiotics is timely access to accurate diagnostic tests. Diagnosis of pneumonia and acute pyelonephritis is challenging as symptoms are often weak and nonspecific and the current methods for focal and etiological diagnosis have low sensitivity and specificity and often deliver results after the antibiotic treatment decision has been made (9, 13, 14).

The COVID-19 pandemic has highlighted this problem. Quick and correct classification of pneumonia as COVID-19, another viral or bacterial pneumonia, or even COVID-19 complicated with bacterial pneumonia, is of vital importance when selecting the correct treatment (including antibiotics), and the correct isolation and infection control measures.

In order to make the correct diagnosis and prescribe a targeted, appropriate treatment within a few hours of admission, it is important to the physician to be able to answer the following three questions: a) Is it an infection that requires antibiotic treatment (*infection marker*)? b) Where is the infection located (*focal diagnosis*)? c) Which bacteria should the prescribed antibiotic treat (*etiologic diagnosis*)?

Infection marker

To diagnose an infection, a systemic response to the infection is required: abnormal temperature, increased leucocyte count or neutrocytosis, or elevated C-reactive protein (CRP). There is some uncertainty associated with CRP because it has a delayed response to bacterial infection and often is elevated in non-infectious inflammatory conditions(15). A more sensitive and specific marker is required that can differentiate between bacterial and viral infection and can reflect the severity of the infection(16). Serum Procalcitonin (PCT) has potential as a diagnostic tool in suspected bacterial infection(17) and can distinguish between viral and bacterial pneumonia(18). SuPAR (soluble urokinase plasminogen activator receptor) might have a potential as a marker for acute bacterial infections requiring antibiotic treatment. However, there are no well-conducted studies which compare these three biomarkers diagnostic abilities for bacterial infections in general or in relation to diagnosis of pneumonia and acute pyelonephritis(16, 19).

The abundant and restricted expression of surfactant protein D (SP-D) within the lung makes this protein a specific marker for lung disease(20). Krebs von den Lungen-6 (KL-6) is expressed in the lung and is a diagnostic and prognostic marker of interstitial lung disease(21-24). The inflammatory glycoprotein YKL-40 is associated with severity of interstitial lung disease(25). The value of these lung injury markers for diagnosing pneumonia needs further investigation.

b) Focal diagnostics

The diagnosis of pneumonia is primarily based on clinical symptoms and findings, supplemented with chest X-ray, which has a low sensitivity and specificity in the diagnosis of pneumonia (26). Identifying an improved imaging alternative with high diagnostic sensitivity and specificity and minimal risk to the patient is imperative. High-resolution CT scan (HRCT) provides a detailed diagnosis of thoracic diseases, but the radiation dose is high and potentially harmful. Low-dose CT scan has shown promising diagnostic results, but the radiation dose is still potentially harmful(27). Ultralow dose CT scan of the thorax (ULDCT) could be an alternative but has yet to be studied within an ED context. Ultrasound scanning (US) of the lungs is useful to diagnose pulmonary edema and pleural effusion, but its value for diagnosing pneumonia needs further investigation(28).

Currently, there are no readily available imaging methods to verify the diagnosis of acute pyelonephritis. The diagnosis is primarily based on unspecific clinical findings (29), and is often not confirmed microbiologically (30). Complicating factors such as hydronephrosis/pyenophrosis and renal abscess can be visualized with conventional US (31). Contrast enhanced US (CEUS) and US with Doppler and spectral-assessment seems to be promising diagnostic imaging modalities of acute renal inflammation (32, 33). The value and suitability in a clinical setting of these more advanced US investigations are unknown.

Etiological diagnostics

Sputum can be cultivated to determine a specific bacterial infection. However, results are often first available after discharge of the patient or completion of treatment (9, 16). Therefore, better and faster methods are required. A so-called 'point-of-care' (POC) tool providing rapid microbiological results on e.g. sputum samples would be beneficial. Systems are available today based on PCR (polymerase chain reaction) methods with results available within one hour for a variety of viral and bacterial agents (34). The impact of such systems has not been investigated in an ED context.

The diagnosis of acute pyelonephritis is verified by significant bacteriuria (30), but up to half of the patients with clinical pyelonephritis fails to meet this diagnostic criterion. Unfortunately, the sample to result time for urine cultures are at last one day if negative – two to three days if positive. (29, 30, 35, 36) Urine test strips are unreliable with low specificity and low predictive values (37). Therefore, a POC tool is necessary, which can provide rapid results and quickly identify the many patients who do not have bacteria in the urine. One tool is urine flow cytometry, which has shown promising diagnostic value with a high negative predictive value for the exclusion of bacteriuria (38). However, better documentation for its use as an ED diagnostic screening method is vital.

Hypothesis, aim and objectives

Our broad hypothesis is that by improving the knowledge of ED infected patients and by introducing new diagnostic tools, which contribute to the development of rapid and more accurate diagnosis of acute infections, a more appropriate antibiotic therapy can be administered.

The project aims to adapt and evaluate alternative diagnostic tools and working methods that support a prompt and accurate diagnosis of hospitalized patients suspected of an urgent infection – with special focus on pneumonia and acute pyelonephritis.

The research objectives are:

1) What is the patient characteristics and course of treatment of the different ED infections?
2) What is the diagnostic value of the infection markers PCT, suPAR, and CRP in patients with suspected pneumonia and pyelonephritis?
3) What is the diagnostic value of the lung injury markers SP-D, YKL-40, and KL-6 on diagnosing pneumonia
4) What is the impact of bedside-US and ULDCT on diagnosing pneumonia ?
5) What is the impact of advanced US on diagnosing pyelonephritis?
6) What is the impact of bedside-US on diagnosing hydronephrosis?
7) What is the impact of POC-PCR analysis of sputum on antibiotic prescriptions?
8) What is the impact of POC urinary flow cytometry on diagnosing and excluding bacteuria?

The goal is to combine the results of all these eight objectives into one novel diagnostic model which the ED physician can apply when patients with suspicion if an infection is admitted.

Method

Study design

The study is designed as a multifaceted multicenter diagnostic study, where participants are offered additional diagnostics depending on the suspected focus of infection (Figure 1).

The study protocol was reported in accordance with the SPIRIT (Standard protocol items: Recommendations for interventional trials) statement(39).

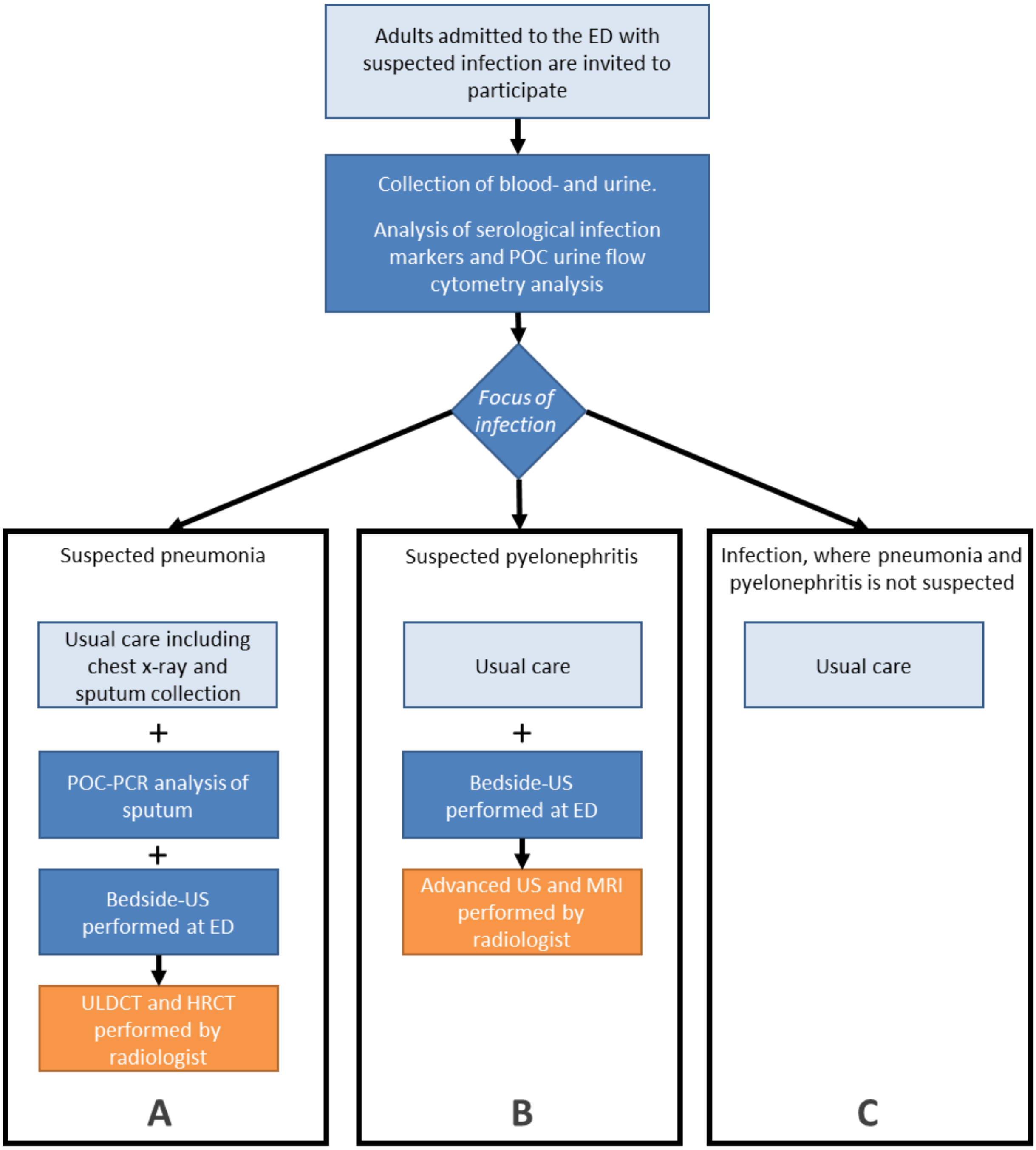

Figure 1 Design of patient flow

Setting

The study will recruit patients from here regional hospitals in the Region of Southern Denmark: the ED in Kolding, Lillebælt Hospital, the ED in Aabenraa, Hospital Sønderjylland, and the ED in Odense, Odense University Hospital. Six study assistants are associated to the study to ensure patient recruitment and data collection. They will be trained and certified in US of lung and kidneys (Objective Structured Assessment of Ultrasound Skills (OASUS) test), sputum collection, operation and integration of POC-PCR as well as POC urine flow cytometry.

Enrollment to the study commences from February 2021, and continues until the predefined sample size has been reached, which is estimated to be no longer than eight months. The last patient is expected to be enrolled in the end of September 2021. The patients will be enrolled from Sunday to Friday 10am-8pm except on holidays.

Population and eligibility criteria

Mentally competent adults admitted to the ED will be invited to participate in the study, if the physician, receiving the patient, suspects the patient has an infection (e.g. indication for blood culture). If the physician at this moment suspects pneumonia, the patient will be offered additional diagnostics showed in track A in Figure 1. The physician will base his/her suspicion on e.g. clinical symptoms such as cough, increased sputum production, chest tightness, dyspnea and fever > 38°C, and indication for chest x-ray (40).

Likewise, if the physician suspect pyelonephritis/urosepsis, the patient will be offered additional diagnostics showed in track B in Figure 1.

The exclusion criteria are:

- If the attending physician considers that participation will delay a life-saving treatment or if patient is transferred directly to intensive care unit (track A, B, and C)
- Patients which have been hospitalized within the last two weeks, to exclude hospital-acquired infections
- Only for patients with suspected pneumonia (track A)
    - Patients < 40 years and patient which have been included in the study once are excluded from the ULDCT and HRCT due to radiation risk
    - Pregnant women due to radiation risk to the unborn child are excluded from the ULDCT and HRCT
    - Patients treated with prednisolone 20 mg/d in the last two days will be excluded from POC-PCR analysis of sputum
- Only for patients with suspected pyelonephritis (track B)
    - Common exclusion criteria for MRI e.g. pacemaker and claustrophobia
    - Patients with known allergy to US contrast
    - Pregnant women due to unknown impact of contrast to the unborn child

Recruitment

Six appointed members of the research group will be study assistants (4 Ph.D. students and 2 one-year research medial students) will identify eligible patients through the local logistic system, and contact the emergency treating physician as early as possible after patient arrival. According to regional guidelines, a clinical assessment is performed within half an hour from arrival by the physician (41). According to the eligibility criterion the physician will assess whether the patient, has a suspected infection and if possible the focus of infection.

If the physician finds the patient eligible for inclusion, the patient will be contacted by a study assistant and informed about the study both verbally and in writing. The information will preferably be held in a patient room without presence of fellow patients. The patient will be offered up to 30 minutes consideration time if needed. The consideration time is set based that it is an acute condition which requires rapid treatment. The patient is allowed to have relatives present during the information and consideration. If the patient agree to participate, consent is obtained, which includes accept to collect blood samples for additional infection markers, collect urine for additional analysis, and collect health related data from the medical record from previous admissions, current admission and readmissions. If pneumonia is suspected, the consent also includes accept to bedside US, ULDCT, HRCT, and additional analysis of sputum. If pyelonephritis is suspected the consent also includes bedside US, advanced US, and MRI. If the patient does not wish to give consent to all parts of the study, consent can be given to individual parts.

The consent will be in writing, dated and signed. A copy of the signed consent will be attached to the participation information and handed out to the patient. For participants in isolation rooms, the consent form will not be collected physically for storage. A picture of the form will be taken and saved electronically in REDCap (Research Electronic Data Capture).

Procedure

The procedure is illustrated in Figure 2. Upon arrival of potential participants, the study assistant contacts the nurse and ask her/him to wait to ordinate routine blood sample collection until the study assistant has invited the patient to participate in the study.

As soon as possible after recruitment (within half an hour from arrival), the study assistant will ordinate blood samples for routine blood culture and standard admission Workpackage (RBC, hemoglobin, monocytes, lymphocytes, WBC, WBC Differential Count, CRP, Sodium, Potassium, Calcium, Albumin, Creatinine, Uric Acid, Alkaline Phosphatase, GGT, Bilirubin, ALAT, LDH, INR, and Glucoses) and project samples.

The samples will be collected by a medical laboratory technologist. Urine sample for routine urine culture and POC urine flow cytometry will also be ordered, and collected by the study assistant. If the participants has suspected pneumonia, a sputum sample will be collected and analysed according to normal procedure. Some of the samples will be further analysed by POC-PCR and linked to a microbiological guidance. The study assistant also collects information about background, habitual and current symptoms through an interview with the patient. Above mentioned steps have to be performed within four hours after arrival.

If the participant has suspected pneumonia or pyelonephritis the study assistant will perform bedside US and ordinate ULDCT/HRCT or advanced US/MRI, respectively. The imaging will be performed within 24 hours after arrival.

During admission and after discharge, clinical, para-clinical and follow-up data will be collected from the medical record by the study assistants. All collected data will be entered in REDCap.

The procedure for the individual steps is described further in the next six subsections.

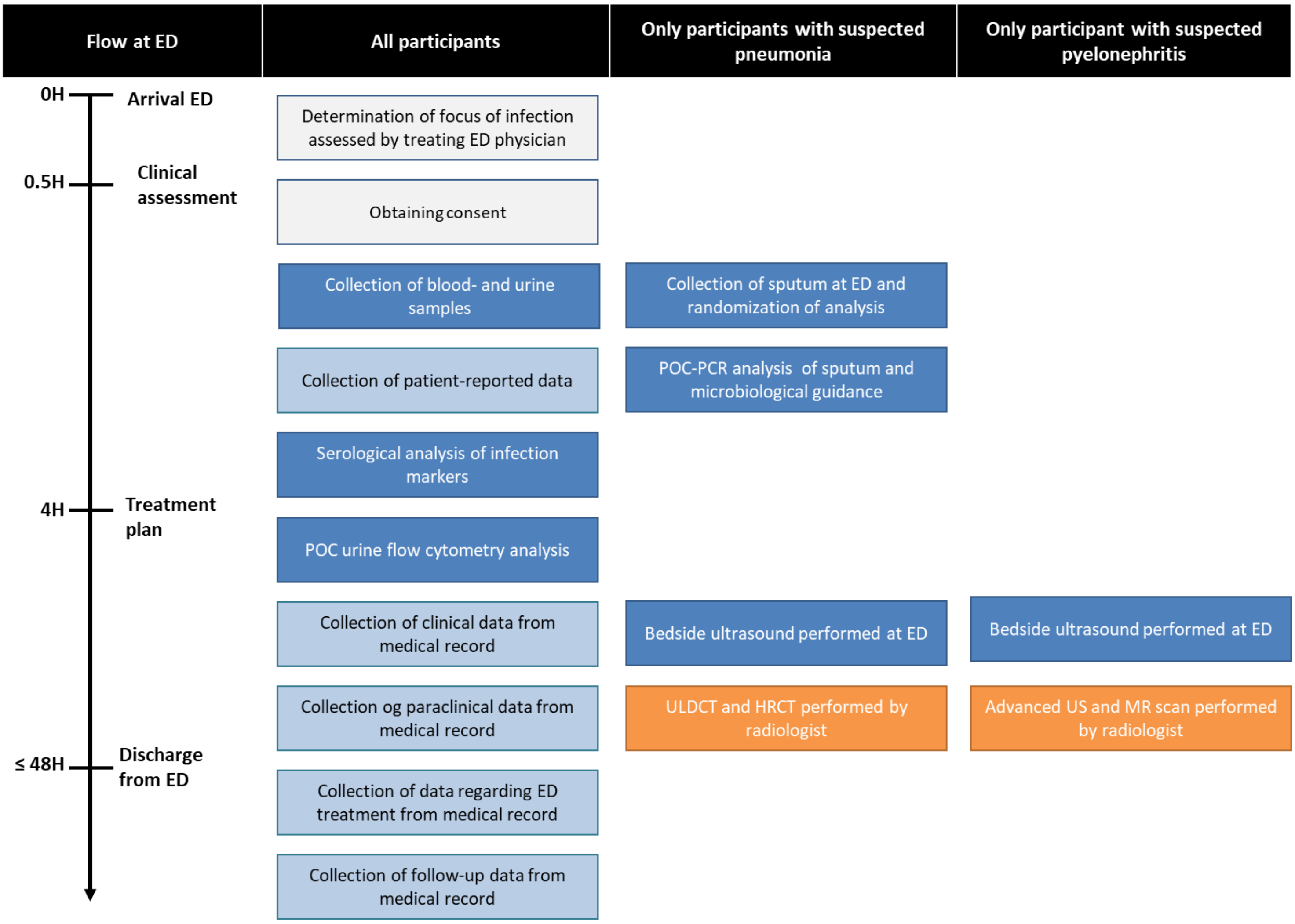

Figure 2 Defined procedure for data collection of enrolled patients

Additional infection markers - PCT and suPAR

The blood will be collected in an 10mL EDTA tube. The samples will be transferred to the local laboratory for analysis. The results will be saved in a study database and not be visible for the physician in the medical journal.

Serum procalcitonin (PCT)

Serum PCT concentration is quantified with an automated sandwich immunoassay "ECLIA" (Elecsys, BRAHMS PCT-analyses) on Cobas e801. Calibration is performed after Cobas e pack has been registered in the instrument and is standardized to the BRAHMS PCT LIA assay. The correlation of Elecsys BRAHMS PCT analyses has been compared to BRAHMS PCT LIA and to BRAHMS PCT sensitive KRYPTOR with similar results of r=0.981 and r=0.988 respectively.

Serum PCT is tested within two hours from the collection of the blood sample. Quality control is performed after each calibration and regularly following the standard procedure. The manufacture states a lower limit of detection 0.02 µg/L up to 100 µg/L. The functional assay sensitivity is identified at ≤ 0.06 ng/mL. In this study a range from 0.06 µg/L to 100 µg/L will be measured. Normal healthy individuals have a PCT concentration < 0.1 µg/L. All plasma samples are screened for potential interfering substances like bilirubin, hemoglobin and lipids and no results will be included with significant interference. There is no hook-effect in PCT concentrations measured up to 1000 µg/L.

The precision of PCT assay is expected to be <3% CV or similar. This is estimated from the internal quality controls using PC PCT1 (lot.419495) and PC PCT2 (lot.419497) at target PCT levels 0.49 and 9.44 ng/L showing a precision of 2.67 % CV and 2.63 % CV, respectively.

Serum soluble urokinase plasminogen activator receptor (suPAR)
Serum suPAR is measured using suPARnostic Tubilatex assay reagents (validated on Cobas c111) protocol for Cobas c702 and c502 applying the Multi-Pack cassettes (Roche Diagnostics, Mannheim, Germany) (42). Calibration is performed at least once a month or in connection to a new batch of TurbiLatex reagents, after calibration a quality control is performed. Serum suPAR is tested within two hours from the collection of the blood sample.

Measure range of the suPARnostic Tubilatex assay is 1.8 µg/L to 16.0 µg/L on Cobas c502 analyzer. The assay's limit of blank, limit of detection and limit of quantification are 1.0 µg/L, 1.2 µg/L and 1.2 µg/L respectively. Expected values for patients attending ED's range from 3-6 µg/L and can reach double digits in patients with severe disease related to poor prognosis. High concentration of SuPAR above 20 µg/L may be false positive results related with interference used by high concentration of hemoglobin, lipids or bilirubin. There is no identified interference in concentrations of bilirubin >350 µmol/L, triglycerides > 3.3g/L, hemoglobin > 1.4 g/L or rheumatoid factor > 440 IU/mL. The highest concentration of suPAR is tested at 47.5 µg/L without hook-effect and the linearity is from 1.8 µg/L to 26.6 µg/L. The mean value of precision of the test is 3.4 µg/L, 7.1 µg/L, 10.2 µg/L for low, middle and high concentrations of SuPAR respectively. The accuracy of suPARnostic Tubilatex is compared with suPARnostic ELISA with similar results < 15 % of difference.

The precision of suPAR assay is expected to be < 5% CV or similar. This is estimated from external quality assessment material, HK 19 (Product code 2226 DK, Lot. No. 201808) analyzed repeatedly during five different days on c502 and c702 and the mean content of suPAR determined by turbidimetry was 2.15 mg/ L and 2.03 mg/L (CV% 4.56 and 5.52) for the Cobas c502 and c702 instruments, respectively.

POC urine flow cytometry
Within 4 hours from arrival, a urine sample will be collected according to routine procedure. The study assistant will be responsible for the collection to optimize compliance. Preferably a mid-ray urine or for catheter users (if possible) by introducing a clean catheter for optimal urine quality. The sample will be divided into two aliquots; one half for routine urine culturing in the Microbiological Department, and one half for POC urine flow cytometry analysis in the Biochemical Department.

The POC urine flow analysis is performed by the Sysmex UF -5000, a fully automated flow cytometry analyser for particle analysis in urine. It discriminates and counts 17 diagnostic parameters of cells and formed elements in urine. Particles are stained by specific flourochromes for nucleic acids and for surface structures, and are exposed to a blue semi-conductor laser-light at 488 nm wavelength. Counting and classification is based on signals of forward scattered light (FSC), side scattered light (SSC), side fluorescent light (SFL), and depolarized side scattered light (DSS). The pattern of individual light signals is translated by algorithms allowing identification and classification into the particle categories, and results will be collected for bacteria, White Blood Cells (WBC), and Red Blood Cells (RBC). The light signals differ for Gram negatives and Gram positives, and this can be recognized by the analyser. For the above mentioned parameters, the analytical performance of the urine flow cytometer will be compared to the gold standard a urine culture on selective agar plates. A protocoled procedure for the collection and handling of samples will ensure consistent data quality.

The analysis will be carried out by laboratory assistants. The results of the POC urine flow cytometry analysis are not currently reckoned state-of-the-art and will be used for scientific purpose only. Results will be coded by the laboratory technician and not visible in the electronic laboratory system.

Additional POC-PCR analysis of sputum if suspected pneumonia

A sputum samples will be collected according to standard procedure as soon as possible after recruitment. The sample will be divided into two aliquots; one for routine sputum culturing by the Microbiological Department, and one for additional analysis.

The sample for additional analysis will be randomly assigned to one of two groups with 1:1 allocation:

1. Control: no POC-PCR will be done and no further intervention will be performed.
2. POC-PCR analysis linked to advice from microbiologist: Sputum samples will be analysed by the study assistant and results will be saved in the local microbiological database. The study assistant will tell a study microbiologist to contact the physician upon performing the 4-hour-treatment plan, and discuss optimal antibiotic treatment plan for the patient based on clinical sign and symptoms, chest X-ray, routine blood analyses and results from POC-PCR analysis.

The POC-PCR used in this study is by Biofire FilmArray Pneumonia Panel plus (Biomérieux, Marcy l'Etoile, France), which is a closed automatic system, nested multiplex PCR, that includes all the steps of molecular diagnostics, with running time of about 75 min, including sample preparation and nuclei-acid extraction. The panel includes 15 typical bacterial pathogens, three atypical bacterial pathogens, nine viruses and seven antimicrobial resistance genes. The results for typical colonizing bacteria are reported semi-quantitatively providing estimates to the nearest whole log as genome copies/ml ranging from 10^4 to 10^7 copies/mL., results from viruses, fungus and atypical bacteria are reported as "detected" or "not detected" and antimicrobial resistance genes are reported qualitatively in the case of simultaneous detection of a compatible pathogen. Testing of specimens using the panel will be conducted in accordance with the manufacturer's instruction (43). Each site will have a pocket laboratory protocol to ensure consistent data collection in quality and safe handling of specimens.

Additional imaging if suspected pneumonia

Bedside US of the lung will be a FLUS (Focused Lung UltraSound), which examines exclusively for life-threatening conditions. FLUS is used to diagnose pneumothorax, pleural effusion and interstitial syndrome. Video clips will be stored during the scan.

ULDCT of thorax will be performed by a radiologist. The patient will be in horizontal rest. The first 10 participants on each site will enter a pilot study to ensure the best possible image quality of ULDCT via customization of the CT, which are different at each hospital. This to ensure uniform quality and dose. A specially designed technical ULDCT protocol has been developed using a breast phantom (N1 'Lungman', Kyoto Kagaku, Japan) equivalent to an 80 kg man. To develop the protocol a GE Revolution CT (GE Healthcare, Waukesha, IL, USA) with 350 mm scan area and 128x0.625mm detector has been used. The ULDCT will be performed with a fixed tube and without a planning image.

As reference standard a High Resolution Computed Thermography HRCT scan of thorax will be taken at the same time as ULDCT on the same scanner. HRCT will be performed according to standard clinical protocols at each hospital, but only doing inspiration to limit radiation.

The three scans will take place within 24 hours after study recruitment. The results of bedside US, ULDCT, and HRCT will be available to the treating physician, when described. If a result requires immediate action, the clinician will be contacted directly by the examiner. If the participant is discharges from the hospital before the scans have been performed, the scan are offered on an outpatient basis.

Additional imaging if suspected pyelonephritis

Bedside US of the kidneys will be performed in order to assess whether hydronephrose is present or not. Video clips will be stored during the scan.

An advanced US will be performed at the Radiology Department, and includes Doppler with spectral assessment and registration of RI and PI values in upper, middle, and lower parts of kidneys. It also includes a CEUS (contrast enhanced US) with intravenous injection of 1.5 mL ultrasound contrast. The advanced US will be performed in order to identify inflammatory changes in the kidneys. Video clips from the advanced US will be stored in the Picture Archiving and Communication System (PACS). Clips form CEUS will furthermore be exported anonymously to an external software application (Vuebox) for post-process evaluation.

As reference standard a MRI of the kidneys will be performed at the Radiology Department at the same time (as close as possible) as the advanced US. The scan will include following sequences: planning, Dixon, T1 mapping,T2, T2 mapping, diffusion ADC (100, 400, 800), MRA (3D VIBE angio), and Phase Contrast.

The three scans will take place within 24 hours after study recruitment. The physician responsible for the treatment of the patient, will be informed of the results of the US scans and if MRI, when described. If a result requires immediate action, the clinician will be contacted directly by the examiner. If the participant is discharges from the hospital before the scans have been performed, the scan are offered on an outpatient basis.

Research biobank

In this study, blood will be collected for a research biobank to store blood until analysis is feasible. Analysis will take place after all samples have been collected.

Biobank blood includes one tube of 10 mL EDTA plasma (suPAR and SP-D) and one tube of 4 mL LiHeparin (YKL-40 and KL-6). All samples will be stored locally in a -80 °C freezer. The samples are pseudonymized with a code to protect the identity of the participants. All samples and code lists are stored safely and separately to prevent unauthorized access.

The expiry date of the research biobank is the last day of recruitment, which is expected to be September 2021. After expiry date, the remaining material in the research bank will be destroyed.

Information from the medical record

The information which will be obtained in the medical record are listed in Table 1.

Table 1 Information to be obtained from the medical record

Time of collection	Variables i medical record	Purpose
Prior ED arrival	Antibiotic treatment within last 6 months	Confunding
Upon ED arrival	Vital signs	Patient characteristic
	Triage	Patient characteristic
	Comobidity	Patient characteristic
	Antibiotic allergy	Patient characteristic
	Inhalation medication	Confounding
During admission	Prescribed antibiotic	Outcome
	Blood test results	Patient characteristic
	Microbiological test results of blood, urine, swab and sputum	Outcome
	Diagnostic imaging results	Outcome
	ICU transfer	Outcome
	Findings regarding diagnosis and treatment described in clinical notes	Outcome and patient characteristic
	Date of discharge	Outcome
After hospital discharge	Date of mortality	Outcome
	Date of readmission	Outcome

	C. diff infection	Outcome

Outcomes and statistical analysis

Before data analysis, data will be pseudonymised. Datamanagement and statistical analysis will be perform in STATA.

Annually, 5.7% of patients are diagnosed with pneumonia and 2.4% with pyelonephritis (data from the ED at Hospital Sønderjylland). Taking into account exclusion criteria, weekends/holidays/missing data and experience in patient recruitment, it is estimated that at least 1000 patients admitted with suspected infection must be included in the study from the three hospitals over a 7-month period, of which at least 250 patients will be diagnosed with pneumonia and at least 150 patients with pyelonephritis.

Patient characteristics and course of treatment

Patient characteristics according to pre-defined clinical parameters will be noted in REDCap. The parameters include symptoms, signs, disease severity, vital signs, triage at arrival, comorbidities, functional status, resident status, antibiotics prescribed during the last 6 months and medical history.

Logistic univariate and multivariate analysis will be carried out for selected risk indicators of pneumonia or pyelonephritis. For pneumonia diagnosis at least 15 variables have to be analyzed, and 200 patients with verified diagnosis are needed (50+15 events/variable). For pyelonephritis at least 10 variables have to be analyzed, and 150 patients with verified diagnosis are needed (50+10 events/variable). (44)

Diagnostic value of infection markers

The concentration of PCT, CRP, and suPAR are evaluated for the verified diagnosis of pneumonia/pyelonephritis and compared to a control group using receiver-operating characteristic (ROC) curves and identifying an optimal cut-off. Secondary outcomes will evaluate which infection marker in combination of severity scores can assess disease severity and predict short-term and long-term mortality in pneumonia patients.

The study is designed to be able to find a difference in Area under the curve (AUC) from 0.7 to 0.8 between two tests, which requires 200 verified pneumonia cases and 200 controls (power 0.8, alpha 0.05, AUC below 0 hypothesis 0.7) and 150 verified pyelonephritis cases and 150 controls (power 0.8, alpha 0.05, AUC below 0-hypothesis 0.6) (45).

Diagnostic value of bedside-US and ULDCT in diagnosing pneumonia

The radiological findings will be described systematically using standardized assessment templates for US, ULDCT and HRCT. The project assistant will describe the bedside US findings, and radiologists will describe the ULDCT and HRCT diagnosis with standardized relevant clinical information. The US operators and the MRI-operator will be blinded to the results of each other. Study consultants with experience in lung imaging will post-process describe the scans and video clips systematically using developed research templates.

A reference diagnose for pneumonia is defined by a panel of experts. The experts are two consultants from Emergency Departments, who individually will determine if the diagnose was pneumoni, or not. The final diagnoses will be based on all available relevant information including e.g. results of imaging, blood sample and clinical information. A standardized template will be used. Disagreement will be discussed until a consensus is reached.

The results from bedside US and ULDCT of thorax are compared with HRCT of thorax and the reference diagnose. The advantages and disadvantages of the diagnostic capability of the individual modalities are described.

It is assumed that the reference diagnosis finds 98% of patients and index test 90%. With a power of 80%, at least 132 patients should be included (one-sided McNemar test).

The primary outcome is sensitivity and specificity, LLR+, LLR-, AUC for diagnosing pneumonia. To test the hypothesis a chi-squard test will be performed.

Secondary outcome will be sensitivity and specificity for chest x-ray. All outcomes will be reported with corresponding confidence intervals when relevant. Secondary analysis will compare sensitivity, specificity, ROC curve and AUC for ULDCT or bedside US compared to chest x-ray, using McNemar Chi-square to test the null-hypothesis

Diagnostic value of bedside US and advanced US in diagnosing pyelonephritis

The radiological findings will be described systematically using standardized assessment templates for bedside US, advanced US, and MRI. The project assistant will describe the bedside US findings, and radiologists will describe the advanced US and MRI findings with standardized relevant clinical information. The US operators and the MRI-operator will be blinded to the results of each other. Study consultants with experience in kidney imaging will post-process describe the scans and video clips systematically using developed research templates.

A reference diagnose for pyelonephritis is defined by a panel of experts. The experts are two consultants from Emergency Departments, who will determine the final diagnoses based on all available relevant information including e.g. results of imaging, blood sample and clinical information. A standardized template will be used. Conflicts will be discussed until consensus is reached.

The results from bedside US and advanced US of kidney are compared with results from the MRI and reference diagnose. The advantages and disadvantages of the diagnostic capability of the individual modalities are described.

It is assumed that the reference diagnose finds 98% of patients and index test 90%. With a power of 80%, at least 132 patients must be included (one-sided McNemar test).

Primary outcome is sensitivity and specificity, LLR+, LLR-, ROC curve and AUC for diagnosing pyelonephritis. To test the hypothesis a chi-squard test will be performed.

Effect of POC-PCR on antibiotic prescription

The antibiotic prescribed in the 4-hour plan, will be collected. The antibiotic type will be classified in four groups; 1) no antibiotic prescription, 2) narrow spectrum antibiotics, 3) broad spectrum antibiotics and 4) broad spectrum antibiotics not targeting pneumonia treatment. The primary outcome is to investigate difference in type of antibiotic prescription in the three randomization groups.

Secondary outcomes include revision of antibiotic treatment at 48 hours, registered adverse events (e.g. lengh of stay, in-hospital mortality, 30-days mortality and clostridium infections), and agreement between POC-PCR results and results of sputum culture.

To achieve a power of 82% for the main analysis, 200 patients with suspected CAP must be included. Generalized mixed effect models will be adjusted for strong predictors. If the sample size is not sufficient for a generalized mixed effect models the corresponding univariate analysis will be conducted

Amendment: Intervention group: sputum samples analysed by POC-PCR. Control group: routine microbiology analysis. It is a superiority randomized trial. Primary outcome is targeted versus non-targeted antibiotic treatment prescribed at four hours after admission. Targeted treatment is defined as narrow spectrum antibiotics directed against CAP, antibiotics directed against a detected respiratory pathogen or no antibiotics (e.g. in the absence of a bacterial pathogen and/or presence of a viral pathogen). Non-targeted treatment is defined as broad spectrum antibiotics not directed against a specific pathogen or antibiotics not directed against CAP. (Appendix VI - Algorithm for antibiotic treatment). The analyses will follow the intention-to-treat principal and a hierarchical mixed effect logistic model will be utilized to analyze the primary outcome to accommodate the hierarchical structure of the random effect, which manifest according to different personnel collecting the samples and geographical variation.

Diagnostic value of POC urine flow cytometry

Common urinary culture shows significant growth of uropathogenic bacterium in approximately 50% of people with suspected pyelonephritis (30). Asymptomatic bacteriuria accounts for about 20% in the elderly population, depending on gender and age (46), which among 1000 inpatients suspected of infection, of which 15% have pyelonephritis, gives a sensitivity of 50% (95% CI: 42-58 %) and a negative predictive value of 90% (95% CI: 77-83%). With the expectation of identifying at least 150 cases of pyelonephritis among 750 inpatients suspected of infection, an improvement in sensitivity to 70% (95% CI: 62-77%) and negative predictive value to 95% (95% CI: 93 -96%) could be found with 95% security.

Ethical considerations and risks

The study is reported to the Scientific Ethics Committee and verbal and written consent for participation from patients is collected. The study are also reported to the Data Inspectorate and registered in ClinicalTrial.com. The study is part of Patient Data Explorative Networking (OPEN). Approval from The Danish Data protection agency for the research biobank has been applied for. Personal information will not be sent abroad.

The collection of blood will be done in relation to planned blood sampling in clinical routine. Blood sampling may cause slight bruising. The collection of urine may be unpleasant. The collection of clinical information may cause nervousness. We will try to minimize these discomforts by engaging with participants about the procedures by the study staff members.

Participation in track A and B will contain additional imaging. Patients under the age of 40 are excluded from the track A due to the extra risk of developing a deadly cancer due to radiation from HRCT. A typical HRCT gives a radiation dose of approximately 2.2 mSv which corresponds to a cancer risk of 1:9100. An X-ray gives a radiation dose of approximately 0.06 mSv which corresponds to a cancer risk of 1:333330. An ULDCT gives a radiation dose of approximately 0.1 mSv which corresponds to a cancer risk of 1:200000. Participation in track A give each participant approximately 2.26 mSv (ULDCT and HRCT) which corresponds to a cancer risk of 1:8850. The examination time of ULDCT and HRCT is approximately 10 minutes.

Patients included in the track B will undergo CEUS and an MRI. The US contrast agent consists of multiple microscopic gas bobbles in a stabilizing shell. These gas bubbles are eliminated with respiration through the lungs. Thus, in contrast to other contrasts used for MRI or CT, it is a non-nefrotoxic substance. Use of US contrast in rare cases cause allergic reactions; less than 1/10.000 exponents require medical treatment due to allergic reaction (47). The examination time of advanced US is approximately 20 minutes. MRI does not provide any radiation dose to the patients. Usual contraindications for MRI also apply in this study, thus we will exclude patients with i.e. claustrophobia or contraindicating metal implanted in the body. The examination time of the MRI is approximately 45 minutes, which the participants are made aware of.

The consent gives the researcher responsible for the study, the sponsor, sponsor's representatives, and control authority direct access to obtain information in the patient medical record. This in order to find data about the patient's health status, which is necessary for the implementation of the study as well as for control purposes, including self-monitoring, quality control, and quality monitoring which is obliged to perform.

The risks and benefits taken into consideration, the overall risk for the participants is minimal, and furthermore, chances are that e.g. the additional diagnostic imaging may inform the clinician in a favorable way before the onset of patient treatment.

Research group

The study originates from the Emergency Research Unit affiliated at the ED at Hospital Sønderjylland and Department of Regional Health Research at University of Southern Denmark, under the leadership of Professor Christian Backer Mogensen and Associate Professor Helene Skjøt-Arkil.

The steering group consists of a representative of the project's research fields, and members are listed in Table 2. The project group consist of clinicians, technicians, project assistants, researchers and leaders in the relevant departments and research fields. The members of the group and their responsibilities including expertise are listed in Table 2. To ensure progress of the involved methods and techniques, four working groups have been established focusing on: 1) biomarkers, 2) focal diagnostics, 3) etiological diagnostics with focus on sputum, and 4) etiological diagnostics with focus on urine. The members of these groups are also listed in Table 2.

Table 2 Members of the project group and their contribution to the project

Position	Name	Hospital#	Contribution
Emergency			
Professor, head of research, and consultant	Christian Backer Mogensen	HS*	Steering group, Research responsible, and expertise in infectious diseases
Associate professor	Helene Skjøt-Arkil	HS*	Steering group, Project responsible, and Working group 1-4
Senior consultant	Matthias Giebner	HS	Head of Department
Senior consultant	Poul Henning Madsen	HL	Head of Department
Physician	Patrick Asbjørn Mikkelsen	HL	Local coordinator
Associate professor and consultant	Stefan Posth	OUH*	Working group 2, and expertise in UL at ED
Infectious disease			
Professor, head of research, and consultant	Isik Somuncu Johansen	OUH*	Expertise in infectious diseases, Working group 4
Respiratory Medicine			
Associate professor and consultant	Christian B. Laursen	OUH*	Working group 2, and expertise in US
Clinical Biochemistry			
Senior consultant	Eva Rabing Brix Petersen	HS	Steering group, Head of Department, working group 1 and 4
Professor and head of research	Ivan Brandslund	HL*	Expertise in flow cytometry and biomarkers, Working group 1+4
Professor and consultant	Jonna Skov Madsen	HL*	Head of Department, Working group 1+4
Chemist	Thor Aage Skovsted	HS	Working group 1 and 4, and expertise in suPAR
Medical laboratory technologist specialist	Morten Andersen	HS	Working group 1 and 4, and project assistant
Radiology			
Associate professor, head of research, and consultant	Ole Graumann	OUH*	Steering group, working group 2, and expertise in US, ULDCT and MRI
Senior consultant	Henrik W. Struckmann	HS	Head of Department
Senior radiography	Pica Ann B. Andersen	HL	Head of Department
Senior consultant	Jakob Møller	HL	Head of Department
Specialist	Bo Mussman	OUH	Expertise in ULDCT, Working group 2
Clinical Microbiology			
Consultant	Flemming Rosenvinge	OUH	Steering group, and working group 3-4
Consultant	Claus Østergaard	HL	Working group 3-4
Senior consultant	Steen Lomborg	HS	Head of Department
Associate professor and consultant	Ming Chen	HS*	Working group 3-4
Study assistants			
Ph.d.-student and physician	Morten Hjarnø Lorentzen	HS*	Working group 2, and data collector
Ph.d.-student and physician	Anne Heltborg	HS*	Working group 2, and data collector

Ph.d.-student, physiotherapist and MSc in Health Science	Mariana B. Cartuliares	HS*	Working group 1 and 3, and data collector
Ph.d.-student and physician	Mathias Amdi Hertz	HS*, OUH	Working group 4, and data collector
Medical student	Jens Juel Specht	HS*	Data collector
Medical student	Frida L. P. Kaldan	HS*	Data collector
Other			
Statistician	Andreas Kristian Pedersen	HS	Statistical analysis and datamanagement

** also affiliated with the University of Southern Denmark, # HS: Hospital Sønderjylland, HL: Hospital Lillebælt, OUH: Odense University Hospital*

Significance of the study

After completion of the study, a novel diagnostic model will be developed. Subsequently, the plan is to test the model in a national setting including at least eight EDs. The results can be implemented in daily work and routines. The Steering group has considerable experience with complex interventions and national studies(3).

The results of the project will have both national and international interest, as the problems are common and the solutions will easily be applied in hospitals with a similar technological context. Securing rapid and reliable diagnosis of two of the most common infections diagnosed in the ED, will encourage the reduction of broad-spectrum antibiotics and thereby the development of multi-resistant bacteria.

The study will also be able to characterize the patients, who are diagnosed at the ED with an infection of unknown origin and prescribed with broad-spectrum antibiotics.

Funding

The study is funded by:

- University of Southern Denmark: 1.650.000kr to cover three years of ph.d.-scholarships
- Region of Southern Denmark: 500.000kr to cover operating expenses
- Hospital Sønderjylland: 4.800.000kr to cover eight years of ph.d.-scholarships
- Research Unit, Hospital Sønderjylland: 500.000kr to cover operating expenses

Funding is deposited in a public account (no: 4597-0010323940) including revision.

The financial sponsors of the study have no representatives in the steering committee and will have no access to or influence on the data, analysis, final results, and publication. The research responsible has furthermore no financial connection to companies or foundations with interest in the study.

Literature references

1. WHO. Antimicrobial resistance - Global report and surveillance. France: World Health Organization; 2014.
2. Bager FE-I, J.; Larsen, AR.; Sönksen, UW. DANMAP 2018 - Use of antimicrobial agents and occurrence of antimicrobial resistance in bacteria from food animals, food and humans in Denmark. 2018.
3. Skjøt-Arkil H, Mogensen CB, Lassen AT, Johansen IS, Chen M, Petersen P, et al. Carrier prevalence and risk factors for colonisation of multiresistant bacteria in Danish emergency departments: a cross-sectional survey. BMJ Open. 2019;9(6):e029000.
4. Sundhedsstyrelsen. Vejledning om ordination af antibiotika. Copenhagen: Danish Health Organisaztion; 2012.
5. Sundhedsstyrelsen. Vejledning om forebyggelse af spredning af MRSA. 2016.
6. Sundhedsstyrelsen. Vejledning og forebyggelse af om spredning af CPO. 2018.
7. Ældreministeriet S-o. National handlingsplan for antibiotika til mennesker - tre målbare mål for en reduktion af antibiotikaforbruget frem mod 2020. 2017.
8. Hellesøe AM, CB.; Anhøj, J.; Jensen, JN.; Bak, H.; Ellermann-Eriksen, S.; Christian, T.;. LKT Antibiotika afslutnings- og evalueringsrapport. 2019.
9. Cartuliares MS, LM.; Gustavsson, S.; Skjøt-Arkil, H.; Mogensen, CB.;. Limited value of sputum culture to guide antibiotic treatment. DMJ. 2020;Accepted for publication.
10. The top 10 causes of death: World Health Organization; 2018 [Available from: https://www.who.int/news-room/fact-sheets/detail/the-top-10-causes-of-death (accessed July 2019).
11. Kennedy M, Joyce N, Howell MD, Lawrence Mottley J, Shapiro NI. Identifying infected emergency department patients admitted to the hospital ward at risk of clinical deterioration and intensive care unit transfer. Acad Emerg Med. 2010;17(10):1080-5.
12. Meehan TP, Fine MJ, Krumholz HM, Scinto JD, Galusha DH, Mockalis JT, et al. Quality of care, process, and outcomes in elderly patients with pneumonia. JAMA. 1997;278(23):2080-4.
13. Chandra A, Nicks B, Maniago E, Nouh A, Limkakeng A. A multicenter analysis of the ED diagnosis of pneumonia. Am J Emerg Med. 2010;28(8):862-5.
14. Reed WW, Byrd GS, Gates RH, Jr., Howard RS, Weaver MJ. Sputum gram's stain in community-acquired pneumococcal pneumonia. A meta-analysis. West J Med. 1996;165(4):197-204.
15. Schuetz P, Christ-Crain M, Muller B. Procalcitonin and other biomarkers to improve assessment and antibiotic stewardship in infections--hope for hype? Swiss Med Wkly. 2009;139(23-24):318-26.
16. Savvateeva EN, Rubina AY, Gryadunov DA. Biomarkers of Community-Acquired Pneumonia: A Key to Disease Diagnosis and Management. Biomed Res Int. 2019;2019:1701276.
17. Hey J, Thompson-Leduc P, Kirson NY, Zimmer L, Wilkins D, Rice B, et al. Procalcitonin guidance in patients with lower respiratory tract infections: a systematic review and meta-analysis. Clin Chem Lab Med. 2018;56(8):1200-9.
18. Self WH, Balk RA, Grijalva CG, Williams DJ, Zhu Y, Anderson EJ, et al. Procalcitonin as a Marker of Etiology in Adults Hospitalized With Community-Acquired Pneumonia. Clin Infect Dis. 2017;65(2):183-90.
19. Masajtis-Zagajewska A, Nowicki M. New markers of urinary tract infection. Clin Chim Acta. 2017;471:286-91.
20. Kuroki YT, H.; Chiba, H.; Akino, T. Surfactant proteins A and D: disease markers. BBA Molecular basis of disease. 1998;1408(2-3):334-45.
21. Ishikawa N, Hattori N, Yokoyama A, Kohno N. Utility of KL-6/MUC1 in the clinical management of interstitial lung diseases. Respir Investig. 2012;50(1):3-13.
22. Yokoyama A, Kondo K, Nakajima M, Matsushima T, Takahashi T, Nishimura M, et al. Prognostic value of circulating KL-6 in idiopathic pulmonary fibrosis. Respirology. 2006;11(2):164-8.
23. Utsunomiya A, Oyama N, Hasegawa M. Potential Biomarkers in Systemic Sclerosis: A Literature Review and Update. J Clin Med. 2020;9(11).
24. Guiot J, Moermans C, Henket M, Corhay JL, Louis R. Blood Biomarkers in Idiopathic Pulmonary Fibrosis. Lung. 2017;195(3):273-80.
25. Jiang L, Wang Y, Peng Q, Shu X, Wang G, Wu X. Serum YKL-40 level is associated with severity of interstitial lung disease and poor prognosis in dermatomyositis with anti-MDA5 antibody. Clin Rheumatol. 2019;38(6):1655-63.
26. Caterino JM, Leininger R, Kline DM, Southerland LT, Khaliqdina S, Baugh CW, et al. Accuracy of Current Diagnostic Criteria for Acute Bacterial Infection in Older Adults in the Emergency Department. J Am Geriatr Soc. 2017;65(8):1802-9.
27. Bourcier JE, Paquet J, Seinger M, Gallard E, Redonnet JP, Cheddadi F, et al. Performance comparison of lung ultrasound and chest x-ray for the diagnosis of pneumonia in the ED. Am J Emerg Med. 2014;32(2):115-8.

28. Orso D, Guglielmo N, Copetti R. Lung ultrasound in diagnosing pneumonia in the emergency department: a systematic review and meta-analysis. Eur J Emerg Med. 2018;25(5):312-21.
29. Claeys KC, Blanco N, Morgan DJ, Leekha S, Sullivan KV. Advances and Challenges in the Diagnosis and Treatment of Urinary Tract Infections: the Need for Diagnostic Stewardship. Curr Infect Dis Rep. 2019;21(4):11.
30. Shallcross L, Gaskell K, Fox-Lewis A, Bergstrom M, Noursadeghi M. Mismatch between suspected pyelonephritis and microbiological diagnosis: a cohort study from a UK teaching hospital. J Hosp Infect. 2018;98(2):219-22.
31. Quaia E, Correas JM, Mehta M, Murchison JT, Gennari AG, van Beek EJR. Gray Scale Ultrasound, Color Doppler Ultrasound, and Contrast-Enhanced Ultrasound in Renal Parenchymal Diseases. Ultrasound Q. 2018;34(4):250-67.
32. Mitterberger M, Pinggera GM, Colleselli D, Bartsch G, Strasser H, Steppan I, et al. Acute pyelonephritis: comparison of diagnosis with computed tomography and contrast-enhanced ultrasonography. BJU Int. 2008;101(3):341-4.
33. Kazmierski B, Deurdulian C, Tchelepi H, Grant EG. Applications of contrast-enhanced ultrasound in the kidney. Abdom Radiol (NY). 2018;43(4):880-98.
34. Huang HS, Tsai CL, Chang J, Hsu TC, Lin S, Lee CC. Multiplex PCR system for the rapid diagnosis of respiratory virus infection: systematic review and meta-analysis. Clin Microbiol Infect. 2018;24(10):1055-63.
35. Long B, Koyfman A. The Emergency Department Diagnosis and Management of Urinary Tract Infection. Emerg Med Clin North Am. 2018;36(4):685-710.
36. Rowe TA, Juthani-Mehta M. Diagnosis and management of urinary tract infection in older adults. Infect Dis Clin North Am. 2014;28(1):75-89.
37. Institut SS. Urinvejsinfektioner: Blærebetændelse og nyrebækkenbetændelse Statens Serum Institut: Statens Seum Institut; 2017 [Available from: https://www.ssi.dk/sygdomme-beredskab-og-forskning/sygdomsleksikon/u/urinvejsinfektioner (Accessed April 2019).
38. Herraez O, Asencio MA, Carranza R, Jarabo MM, Huertas M, Redondo O, et al. Sysmex UF-1000i flow cytometer to screen urinary tract infections: the URISCAM multicentre study. Lett Appl Microbiol. 2018;66(3):175-81.
39. Chan AW, Tetzlaff JM, Altman DG, Laupacis A, Gotzsche PC, Krleza-Jeric K, et al. SPIRIT 2013 statement: defining standard protocol items for clinical trials. Ann Intern Med. 2013;158(3):200-7.
40. Sygehusmedicin RfAaD. Baggrundsnotat for hensigtsmæssig anvendelse af antibiotika ved nedre luftvejsinfektioner i almen praksis og på hospital: Rådet for Anvendelse af Dyr Sygehusmedicin; 2016 [Available from: http://www.regioner.dk/media/3996/bgn-antibiotika-nedre-luftvejsinf-vers-1-0-november-2016-267967.pdf.
41. The Region of Southern Denmark traw. Den Regionale Antibiotikagruppe. Diagnostik og behandling af akutte infektioner på sygehusene i Region Syddanmark. 2016.
42. Skovsted TA, Petersen ERB, Fruekilde MB, Pedersen AK, Pielak T, Eugen-Olsen J. Validation of suPAR turbidimetric assay on Cobas (c502 and c702) and comparison to suPAR ELISA. Scand J Clin Lab Invest. 2020;80(4):327-35.
43. BioFire. 2018. FilmArray Pneumonia panel instruction booklet RFIT-ASY0144/145. BioFire SLC, UT.
44. Vittinghoff E, McCulloch CE. Relaxing the rule of ten events per variable in logistic and Cox regression. Am J Epidemiol. 2007;165(6):710-8.
45. Biosoft. easyROC: a web-tool for ROC curve analysis [1.3.1:[Available from: http://www.biosoft.hacettepe.edu.tr/easyROC/ (Accessed January 3rd 2020).
46. Mody L, Juthani-Mehta M. Urinary tract infections in older women: a clinical review. JAMA. 2014;311(8):844-54.
47. Chong WK, Papadopoulou V, Dayton PA. Imaging with ultrasound contrast agents: current status and future. Abdom Radiol (NY). 2018;43(4):762-72.
