# Supplementary material for: Evaluation of point-of-care multiplex polymerase chain reaction in guiding antibiotic treatment of patients acutely admitted with suspected community-acquired pneumonia in Denmark: A multicentre randomised controlled trial
Source: PLoS Med. 2023 Nov 28;20(11):e1004314. doi: 10.1371/journal.pmed.1004314 (PMC10684013; doi:10.1371/journal.pmed.1004314)
Supplement: S3 Text — (PDF) [file pmed.1004314.s008.pdf]

Written consent form Informed consent to participate in a health science project

Project titel: *Infectious Diseases in Emergency Departments (INDEED study)*

Statement from the participant:
I have received written and verbal information, and I know enough about the purpose, methods, benefits, and risks to agree to participate.

I understand that participation is voluntary, and I can withdraw my consent at any time without losing my current or future treatment rights.

I hereby consent to participate in the project and have received a copy of this consent form and a copy of the written information about the project for my own use.

Participant's name: ____

Date: _______________ Signature: ____

If new significant health information arises about you during the research project, you will be informed. If you wish **to opt-out** of receiving information about new significant health information that arises during the research project, please mark here: __________ (place an X)

Statement from the information provider:

I certify that the participant has received both verbal and written information about the trial.

In my opinion, sufficient information has been provided to make a decision about participation in the trial.

Name of the information provider

Date: _______________ Signature: ____

Informed consent

Participant information to individuals admitted acutely with suspected pneumonia

Improved diagnosis of acute infections

Infectious Diagnostics in Emergency Departments (INDEED study)

A collaborative project at Emergency Departments from Hospital Sønderjylland, Hospital Lillebælt, and Odense University Hospital

We would like to ask if you would participate in this study

The project aims to improve the diagnosis of pneumonia in the Emergency Department so that targeted treatment can be initiated as quickly as possible.

Before deciding whether you want to participate in the project, you should fully understand what the project is about and why we are conducting it. Therefore, we ask you to read this information thoroughly.

If you decide to participate, we will ask you to sign a consent form. Remember that you have the right to consult with family, friends, or acquaintances. You also have the right to take your time before signing, but as this is an acute infection requiring rapid treatment, we ask that you make your decision within 30 minutes.

Participation in the project is voluntary. You can withdraw your consent to participate at any time, without giving a reason, and it will not affect your further treatment.

Project objectives

The tools and tests available today for diagnosing pneumonia have many limitations. This challenges reaching a reliable diagnosis quickly and initiating targeted treatment. This can have consequences for hospitalization. Treatment with broader spectrum antibiotics contributes to the development of bacterial resistance.

Therefore, the project aims to find better tools to help the physician make a reliable diagnosis for individuals acutely admitted with suspected pneumonia.

What the project will investigate

The project will investigate:

- Symptoms, signs, and conditions that characterize pneumonia and the severity of the disease
- Markers of infection in the blood that best identify pneumonia and degree of severity
- Usefulness of a new method for measuring bacteria
- Usefulness of identifying bacteria in lung secretions
- Applicability of ultrasound examination and CT scanning with very low radiation risk to diagnose pneumonia

Project Plan

The project involves the Emergency Departments in Aabenraa (Sygehus Sønderjylland), Kolding (Sygehus Lillebælt), and in Odense (Odense University Hospital). From February 2021 to the February2022, 500 adult individuals acutely admitted with suspected pneumonia to the three emergency departments will be invited to participate.

After hospital admission, the staff will approach you and provide information about the project and invite you to participate in the project. Since it is important that an acute infection is treated quickly, we ask you to make a decision within half an hour.

Your involvement in the project

Participation in the project means that you will receive the normal treatment offered by the department but may undergo additional tests.

We will ask you some questions about your symptoms, past and current illnesses, and how you are feeling. In this regard, we request access to your patient record to follow up on any previous admissions, the current admission, and any admissions within the next month after this discharge.

We will collect an additional 14 mL of blood, equivalent to 2 extra tubes, whilst you have blood drawn, and assist you in providing a urine sample.

Of the lung secretions collected as part of the standard treatment, we will take a small portion and examine them using a new method.

The blood, urine, and lung secretions obtained for the project will be destroyed when the project is completed.

If you choose to participate, you will need to undergo two additional lung scans: 1) An ultrasound scan performed in the emergency department, which takes 5 minutes. 2) A CT scan consisting of a scan with very low radiation risk and a high-resolution CT scan, which is the most precise scan used for the lungs today. The CT scan will take a total of 10 minutes.

Your consent will give the principal investigator, the sponsor, and their representative direct access to relevant health information in your electronic health record to carry out, monitor, and control data for the project. In the project, the management of personal data will adhere to data protection laws and regulations. After collecting the desired information, your personal data will be removed from our registration system, and your personal number will be replaced with a code (pseudonymization).

Side Effects, Risks, Complications, and Disadvantages

There are no or very few known risks or side effects associated with participating in the project. All sampling methods are well-known and we have extensive experience in these commonly used procedures. However, there may be risks associated with the tests that we are unaware. Therefore, we ask you to report any problems related to sampling or tests. If we discover side effects that we have not informed you about, you will be informed immediately, and will need to decide whether you want to continue.

The additional blood samples for the project will be drawn at the same time as the blood samples taken upon admission. Risks and side effects of having a blood sample taken can include discomfort, slight pain, and/or bruising, and in some cases, fainting. In rare cases, a minor blood collection or inflammation may occur at the injection site.

The urine sample will be collected in a container during a toilet visit. If you are catheterized or unable to urinate due to illness, assistance may be necessary with urination with a catheter (a thin plastic tube inserted into the bladder through the urethra). This procedure may cause slight discomfort and brief minor bleeding from mucous membranes.

There is no pain associated with the extra scans, but you may experience discomfort during the transfer to the CT scanner. The main risk associated with participation in the project is the additional radiation dose from the CT scan. The extra radiation dose you are exposed to is slightly less than the background radiation you are normally exposed to in one year. The radiation from the scan carries a slight increased risk of cancer development, approximately 0.01-0.1%. This is equivalent to raising the overall lifetime risk of cancer from 25% to 25.1%. However, this risk is considered insignificant in relation to the other risks associated with your current admission.

Your Test Results

If you wish to receive results for the standard blood and urine tests, you can view them on www.sundhed.dk . Results for the additional blood and urine tests in the project will not be available as we do not yet know the significance of the results. If the results have an alarming outcome, the treating physician will be notified, and they will assess how your treatment is affected. The results of the additional examination of lung secretions will be communicated to you by the treating physician.

Scan results will be available to the doctors treating you and will be used as part of their assessment and treatment. If we discover anything that could raise suspicion of other diseases (e.g., cancer), we will contact you through your doctor and offer further examination. If the scans do not provide information that changes your treatment or diagnosis, you will not receive further information about these scans.

Benefits of the Project

The project is essential for improving the hospitalization process for individuals admitted acutely with suspected pneumonia. The project will have a significant impact on the practices in emergency departments and, most likely, the type of antibiotics prescribed by physicians. More targeted antibiotic use will contribute to reducing bacteria resistance to antibiotics, ensuring that infections in the future can still be treated with antibiotics.

For you personally, participation will not immediately affect your treatment process. However, if there are any special complications related to your lung disease, it is more likely we will recognize them more quickly through the additional scans.

Exclusion from the Study

You will be excluded from parts of the project if any of the tests fail due to technical reasons or if your treating physician determines that it is too risky for you to participate.

Access to the Project's Results

The project's overall results will be published in scientific journals throughout 2023- 2024 as well as on the hospital websites. Results relevant to decision-makers in the healthcare sector will be published in Danish media and journals. It is guaranteed that no participants will be identifiable in published materiale. If you are interested in learning more about the project's results, you can access them through http://www.sygehussonderjylland.dk/wm521282 after they are publicly available.

We hope that with this information, you have gained sufficient insight into what it means to participate in the project and that you feel prepared to make a decision about your potential participation. If you would like to know more, please feel free to contact us. Information about your rights are attached at the end of the document (Appendix 1).

If you decide to participate in the project, we will ask you to sign the consent form. You can choose whether to consent to the entire project or only parts of the project. Participation in the project is voluntary, and you can withdraw your consent at any time without giving a reason. It will not affect your ongoing treatment.

Additional information can be obtained by contacting

Professor and chief physician Christian Backer Mogensen
Fælles Akutmodtagelsen, Sygehus Sønderjylland
Kresten Philipsens Vej 15 - 6200 Aabenraa
Christian.Backer.Mogensen@rsyd.dk
Tlf: 79971123

Initiators of the project

The project was primarily developed in collaboration between the Emergency Department, the Biochemical Department, and the Microbiological Department, and the Radiology Department at Hospital Sønderjylland, Hospital Lillebælt, and Odense University Hospital. The project is based at Hospital Sønderjylland and the Institute for Regional Health Research at the University of Southern Denmark, which are responsible for applications and grants.

Financial support for the project

The project has received financial support in the form of Ph.D. students from the University of Southern Denmark (DKK 1,650,000), Ph.D. students from Hospital Sønderjylland (DKK 4,800,000), and operational support from the Region of Southern Denmark (DKK 500,000). The trial administrators have no financial ties to sponsors or other stakeholders in the trial. There will be no financial compensation for patients participating in the project.

Appendix 1: Participant Rights in a Health Science Research Project

As a participant in a health science research project, you should know that:

- Your participation in the research project is entirely voluntary and can only occur after you have received both written and verbal information about the research project and signed the consent form.
- You can withdraw your consent to participate at any time, either verbally, in writing, or through any other clear expression, and withdraw from the research project. If you withdraw your consent to participate, it will not affect your current or future treatment or any other rights you may have.
- You have the right to bring a family member, friend, or acquaintance to the information discussion.
- You have the right to take time to consider before signing the consent form.
- Information about your health conditions, strictly private matters, and other confidential information about you that emerges in connection with the research project is subject to confidentiality. The processing of information about you, including information in your blood tests and tissues, will be done in accordance with the rules of the General Data Protection Regulation, the Data Protection Act, and the Health Act. The data controller in the trial should provide you with more information about your rights under data protection rules.
- There is an opportunity to access research protocols under the Public Access to Information Act. This means that you can access all documents related to the organization of the trial, except for those parts containing trade secrets or confidential information about others.
- You can complain or seek compensation under the rules of the Act of the Complaint and Compensation Procedure within the Healthcare System. If any harm occurs during the trial, you can contact the Danish Patient Compensation Association; see more at www.patienterstatningen.dk.

This supplement was prepared by the Scientific Ethical Committee System and can be attached to the written information about the health science research project. Questions about a specific project should be directed to the trial administrator. General questions about the rights of trial participants can be directed to the committee that approved the project.
