# Supplementary material for: Evaluation of point-of-care multiplex polymerase chain reaction in guiding antibiotic treatment of patients acutely admitted with suspected community-acquired pneumonia in Denmark: A multicentre randomised controlled trial
Source: PLoS Med. 2023 Nov 28;20(11):e1004314. doi: 10.1371/journal.pmed.1004314 (PMC10684013; doi:10.1371/journal.pmed.1004314)
Supplement: S4 Text — (PDF) [file pmed.1004314.s009.pdf]

Text S4: Action card

Guidance of results from POC-PCR
FilmArray Pneumonia Panel plus

This guidance is developed to the INDEED-study (Infectious diseases in Emergency Department).

Emergency department physicians from Hospital Sønderjylland in Aabenraa, Hospital Lillebælt in Kolding, and Odense University Hospital in Odense, will receive this action card along with the results from sputum sample analyses.

In case of doubt in the interpretation of the results, the physician is encouraged to contact the local clinical microbiologist.

<table>
<tr><th rowspan="2">Agents</th><th rowspan="2">Association with CAP#</th><th rowspan="2">Remarks</th><th colspan="2">Antibiotics</th></tr>
<tr><th>First choice</th><th>Penicillin allergy</th></tr>
<tr><td>Streptococcus pneumoniae*</td><td>Frequent and likely pathogen</td><td rowspan="2">Part of the normal microbiota in upper respiratory tract.
May be contamination with pharyngeal microbiota.</td><td>Benzylpenicillin 1.2g (2 mill.IE) x4 i.v.
or
Phenoxymethylpenicillin 0.6g (1 mill.IE) x4 oral</td><td>Cefuroxime 1.5g x 3 i.v.
or
Roxithromycin 300mg x1 oral</td></tr>
<tr><td>Haemophilus influenza*</td><td>Frequent and likely pathogen</td><td>Ampicillin 2g x4 i.v.
or
Benzylpenicillin 1.2g (2 mill. IE) x4 i.v.
or
Piv-ampicillin 1g x3 oral
or
Amoxicillin 1g x3 oral</td><td>Cefuroxime 1.5g x 3 i.v.
or
Doxycycline 100mg x2 first 24 hours oral followed by 100mg x1 oral</td></tr>
<tr><td>Streptococcus pyogenes*</td><td>Probable, but rare pathogen</td><td rowspan="4">Part of the normal microbiota in upper respiratory tract.
These pathogens relatively often represent contamination with pharyngeal microbiota.

Infection caused by Streptococcus pyogenes or Staphylococcus aureus will usually results in severe pneumonia.</td><td>Benzylpenicillin
1.2g (2 mill. IE) x4 i.v.</td><td>Cefuroxime 1.5g x3 i.v.</td></tr>
<tr><td>Streptococcus agalactiae*</td><td>Rare pathogen in adults</td><td>Benzylpenicillin 1.2g (2 mill. IE) x4 i.v.</td><td>Cefuroxime 1.5g x3 i.v.</td></tr>
<tr><td>Staphylococcus aureus*</td><td>Probable, but rare pathogen</td><td>Cloxacillin 1g x4 i.v.</td><td>Cefuroxime 1.5g x3 i.v.</td></tr>
<tr><td>Moraxella catarrhalis*</td><td>Probable pathogen</td><td>Piperacillin-tazobactam
4/0.5g x3 i.v.
or
amoxicillin-clavulanic acid 500/125mg x3 oral</td><td>Cefuroxime 1.5g x3 i.v.
or
Roxithromycin 300mg x1 oral
or
Azithromycin 500mg x1 oral</td></tr>
<tr><td colspan="5"></td></tr>
<tr><td>Legionella pneumophila
Mycoplasma pneumonia</td><td>Likely causative pathogen</td><td>Is not a part of the normal respiratory microbiota.</td><td colspan="2">Azithromycin 500mg x1 i.v./oral</td></tr>
<tr><td>Chlamydia pneumoniae</td><td>Probable causative pathogen</td><td>Is not a part of the normal respiratory microbiota</td><td colspan="2">Azithromycin 500mg x1 i.v./oral</td></tr>
</table>

		Will usually cause mild infections. In case of severe infection, other pathogens/super-infection should be considered.	

Agens	Association with CAP#	Remarks	Antibiotics
*Pseudomonas aeruginosa**<br>*Acinetobacter calcoaceticus-baumannii complex**<br>*Enterobacter cloacae**<br>*Escherichia coli**<br>*Klebsiella (Enterobacter) aerogenes**<br>*Klebsiella oxytoca**<br>*Klebsiella pneumoniae group**<br>*Proteus spp.**<br>*Serratia marcescens**	Very rare causative pathogens	These findings usually represents colonization.	These findings should typically not lead to adjustment of empirical antimicrobial treatment.
Influenza A<br>Influenza B	Frequent pathogens	Is not a part of the normal respiratory microbiota Bacterial superinfection can occur.	Consider whether the patient's pneumonia symptoms can be explained by viral infection, and whether antibiotic treatment is necessary / indicated.
Parainfluenza virus<br>Respiratory Syncytial<br>Adenovirus<br>Coronavirus<br>*(does not include SARS-CoV-2)*<br>Human Rhinovirus/Enterovirus<br>Human Metapneumovirus	Probable pathogens	Usually causes mild infections. In case of severe infection, other pathogens / superinfection should be considered.<br>May be an accidental finding due to previous /recent / asymptomatic infection.	
Not detected<br>*(POC-PCR(FilmArray) is negative)*	A negative result does not rule out pneumonia, but means that CAP caused by the most common pathogens is less likely. Consider whether the pneumonia diagnosis is correct and consider investigation for rare causes of pneumonia (e.g. tubeculosis or *Chlamydia psittaci*).		

#CAP: Community-Acquired Pneumonia

*: concentration (copies/mL) is reported in the POC-PCR (FilmArray) result

Most bacterial causative pathogens of CAP are also part of the normal respiratory microbiota or may colonize the upper respiratory tract, and the clinical relevance of these findings must always be assessed carefully.

For the bacterial agents marked with "*", a concentration (copies/mL) is reported in the POC-PCR (FilmArray) result. There is a reasonable correlation between copies/mL and the culture-based measure "CFU/mL", however, "copies/mL" is typically a factor of 10-100 higher than the corresponding "CFU/mL".

The limits of significance are not well established and depend probably on the agent, the quality of the sample and the clinical context - and must therefore be used with caution. The Infectious Diseases Society of America and the American Society of Microbiology[1] propose the following culture-based limits for hospital-acquired pneumonia:

Culture-based measure	POC-PCR (FilmArray) concentration	Interpretation (caution)
$< 10^4$ CFU/mL	≈ $< 10^5$ copies/mL	Indicates mixture with normal flora
$10^4 – 10^5$ CFU/mL	≈ 10^5-10^6 copies/mL	Gray zone
$> 10^5$ CFU/mL	≈ $>10^6$ copies/mL	Indicates real findings

Developed by microbiologist Flemming Rosenvinge, Department of Clinical Microbiology, Odense University Hospital in Odense, and microbiologist Claus Østergaard, Department of Clinical Microbiology, Hospital Lillebælt in Kolding, Denmark

Version 1.1 – February 7th 2021

[1] *Miller, J. M., Binnicker, M. J., Campbell, S., et al. A Guide to Utilization of the Microbiology Laboratory for Diagnosis of Infectious Diseases: 2018 Update by the Infectious Diseases Society of America and the American Society for Microbiology. Clinical Infectious Diseases, 67(6), e1–e94. https://doi.org/10.1093/cid/ciy381*
