# Supplementary material for: Evaluation of point-of-care multiplex polymerase chain reaction in guiding antibiotic treatment of patients acutely admitted with suspected community-acquired pneumonia in Denmark: A multicentre randomised controlled trial
Source: PLoS Med. 2023 Nov 28;20(11):e1004314. doi: 10.1371/journal.pmed.1004314 (PMC10684013; doi:10.1371/journal.pmed.1004314)
Supplement: S5 Text — (PDF) [file pmed.1004314.s010.pdf]

Data availability and data sharing plan

Anonymized personal data is not subject to data protection legislation by the General Data Protection Regulation (GDPR) in the EU and is therefore allowed to be publicly shared. However, the personal data underlying the results in the article is not possible fully anonymize and is therefore covered by § 10 of the Danish Data Protection Act.

When personal data covered by Section 10 of the Data Protection Act (also applies to pseudonymized information) wishes to be passed on with a view to publication in a recognized scientific journal, it requires permission from the Danish Data Protection Authority, cf. Section 10, subsection of the Data Protection Act. 3, No. 3. However, the Danish Data Protection Authority can only approve this sharing if there is an authority in the informed consent from the ethical approval cf. Section 2, subsection 10 of the Danish Committees Act. In the ethical approval, S-20200188 underlying this project is it stated that personal data is anonymized upon publication. It is, therefore, not possible to share pseudonymized information unrestricted.

Upon request, the project sponsor Christian Backer Mogensen can apply to the Regional Committee for Health Research Ethics, Southern Denmark for an additional supplement of the protocol. This ethical protocol supplement will explain and describe the reason for transferal of the project's personal data to a third party without consent. Such a request can be sent to fortegnelsen-SHS@rsyd.dk marked attention special consultent Signe Bek Sørensen, Kresten Philipsensvej 15, 6200 Aabenraa, Denmark."

**Data sharing statement**	
Will individual deidentified participant data (including data dictionaries) will be shared	Upon request
What data in particular will be shared	Pseudonymized participant data that underlie the results reported in this article, after deidentification (text, tables, figures, and supplementary material).
What other documents will be available	Study protocol, Statistical Analysis plan, Informed consent form.
When will data be available	Up to five years after article publication.
With whom	Investigators and researchers whose proposed use of the data has been approved by an independent review committee identified for this purpose.
For what types of analyses	To achieve the aims in the approved proposal and for individual participant data meta-analysis.
By what mechanism will the data be available	Proposals should request the data to Fortegnelsen-SHS@rsyd.dk where the data will be available for 5 years.
